# OsCAMTA3 Negatively Regulates Disease Resistance to *Magnaporthe oryzae* by Associating with OsCAMTAPL in Rice

**DOI:** 10.3390/ijms25095049

**Published:** 2024-05-06

**Authors:** Shibo Yu, Shengping Li, Wei Wang, Dingzhong Tang

**Affiliations:** State Key Laboratory of Ecological Control of Fujian-Taiwan Crop Pests, Key Laboratory of Ministry of Education for Genetics, Breeding and Multiple Utilization of Crops, Plant Immunity Center, Fujian Agriculture and Forestry University, Fuzhou 350002, China; shibo_fafu@163.com (S.Y.); lishun1981@126.com (S.L.)

**Keywords:** plant immunity, rice blast, OsCAMTA3, OsCAMTAPL, OsALDH2B1

## Abstract

Rice (*Oryza sativa*) is one of the most important staple foods worldwide. However, rice blast disease, caused by the ascomycete fungus *Magnaporthe oryzae*, seriously affects the yield and quality of rice. Calmodulin-binding transcriptional activators (CAMTAs) play vital roles in the response to biotic stresses. In this study, we showed that OsCAMTA3 and CAMTA PROTEIN LIKE (OsCAMTAPL), an OsCAMTA3 homolog that lacks the DNA-binding domain, functioned together in negatively regulating disease resistance in rice. OsCAMTA3 associated with OsCAMTAPL. The *oscamta3* and *oscamtapl* mutants showed enhanced resistance compared to wild-type plants, and *oscamta3/pl* double mutants showed more robust resistance to *M. oryzae* than *oscamta3* or *oscamtapl*. An RNA-Seq analysis revealed that 59 and 73 genes, respectively, were differentially expressed in wild-type plants and *oscamta3* before and after inoculation with *M. oryzae*, including *OsALDH2B1*, an acetaldehyde dehydrogenase that negatively regulates plant immunity. OsCAMTA3 could directly bind to the promoter of *OsALDH2B1*, and *OsALDH2B1* expression was decreased in *oscamta3*, *oscamtapl*, and *oscamta3/pl* mutants. In conclusion, OsCAMTA3 associates with OsCAMTAPL to regulate disease resistance by binding and activating the expression of *OsALDH2B1* in rice, which reveals a strategy by which rice controls rice blast disease and provides important genes for resistance breeding holding a certain positive impact on ensuring food security.

## 1. Introduction

Plants have evolved intertwined immune networks to enhance pathogen resistance [1]. Pattern recognition receptors (PRRs) can recognize pathogen-/damage-associated patterns (PAMPs or DAMPs) and initiate pattern-triggered immunity (PTI), which is crucial for broad-spectrum resistance in plants [2]. The succeeding pathogens secrete effectors to inhibit PTI, and then plants evolve nucleotide-binding domain leucine-rich-repeat-containing receptors (NLRs) to recognize effectors and trigger the second line of defense, which is called effector-triggered immunity (ETI) [3]. PTI is necessary for the full activation of ETI during pathogen infection, and ETI can rapidly increase the expression of key components of PTI [4,5]. Upon the perception of PAMPs or DAMPs, serious defense responses are activated, including an influx of calcium ions (Ca^2+^), oxidative bursts, the activation of mitogen-activated protein kinase, callose deposition, phytoalexin production, and pathogenesis-related (*PR*) gene expression [3,6,7,8]. The influx of Ca^2+^ is one of the earliest coping mechanisms with pathogens in plants [9,10,11]. Ca^2+^ sensor proteins, including calmodulin (CaM) [12,13,14], calcium-dependent protein kinase (CPK) [15,16], calmodulin-like proteins (CMLs) [17,18], calcineurin B-like proteins (CBLs) [19], and CaM-binding protein (CBP) [20,21], can sense changes in Ca^2+^ concentration with their high-affinity Ca^2+^ binding EF hands and play a significant role in immunity [22,23,24,25]. As transcription factors, calmodulin-binding transcription activators (CAMTAs) interact with CaM to regulate plant growth and the response to various abiotic or biotic stresses [26,27]. CAMTAs contain an N-terminal DNA-binding domain (CG-1), TIG-ANK repeats, and a varying number of IQ calmodulin-binding motifs. The CG-1 domain can directly bind to cis-elements ([G/A/C]CGCG[C/G/T]) and regulate downstream immune signals [28]. NtER1, which is highly expressed in flowers and is vital for early ethylene upregulation, was the first reported CAMTA member [29]. In Arabidopsis, there are six CAMTAs. AtCAMTA1 regulates the stress response by maintaining the osmotic balance of cells and negatively regulating cell propagation and differentiation to decelerate cell death and senescence [30]. AtCAMTA1, AtCAMTA2, and AtCAMTA3 (also known as SIGNAL RESPONSTIVE1 [AtSR1]) function redundantly in plant immunity by repressing salicylic acid (SA) levels and the SA-mediated immune pathway [31,32]. The *atcamta3-1* single mutant shows autoimmunity and stunted growth phenotypes [25], while the *atcamta2/3* double mutant and the *atcamta1/2/3* triple mutant display much more extreme autoimmunity [27,33]. Further studies have shown that the transcription of *ISOCHORISMATE SYNTHASE1* (*ICS1*), *CALMODULIN BINDING PROTEIN60-LIKEg* (*CBP60g*), *SAR DEFICIENT1* (*SARD1*), *ENHANCED DISEASE SUSCEPTIBILITY1* (*EDS1*), and *PHYTOALEXIN DEFICIENT4* (*PAD4*), which are positive regulators of the SA pathway and plant immunity [34,35,36], is upregulated in *atcamta3*, *atcamta2*/*3*, and *atcamta1*/*2*/*3* mutants [37,38]. It has also been confirmed that *atcamta3* can repress the expression of *AtEDS1* by binding to its promoter. The loss of function of AtEDS1 in *atcamta3* can rescue elevated SA levels and constitutive disease resistance [25]. The *atcamta3-3d* mutant is a gain-of-function mutant, and both AtCAMTA3 overexpression plants and *atcamta3-3d* mutants exhibit reduced basal immunity against pathogens [32,39]. Moreover, AtCAMTA3 can bind to the promoter of *NONEXPRESSOR OF PATHOGENESIS-RELATED GENES1* (*AtNPR1*), an SA receptor [40,41], and regulate the expression of *AtNPR1* [42]. AtCAMTA3 negatively regulates not only the SA pathway but also the biosynthesis of pipecolic acid (Pip) and N-hydroxypipecolic acid (NHP) by modulating the expression of *AGD2-LIKE DEFENSE RESPONSE PROTEIN 1* (*ALD1*) [38] and *AtCBP60g*/*AtSARD1*, respectively [27,32,37]. Furthermore, cold temperature inhibits the ability of AtCAMTA3 to induce gene expression, and high temperature increases the temperature-mediated susceptible immune response by regulating SA-related genes, such as *AtPR1*, *AtICS1*, *AtNPR1*, and *AtEDS1* in *atcamta3* [26,43]. The *atcamta3* phenotypes can also be rescued by the mutation of two *NLR* genes, *AtDSC1* and *AtDSC2*. The cell death triggered by AtDSC1 or AtDSC2 in *Nicotiana benthamiana* could be repressed by coexpressing with AtCAMTA3, suggesting that AtCAMTA3 may act as a guardee of AtDSC1 and AtDSC2 [44].

Rice is one of the most important staple crops and feeds more than half of the world’s population. Pathogen infection causes enormous losses in rice quality and yield [45], and rice blast disease is the most widespread and serious disease of cultivated rice. In severe disease regions, the losses can even reach 30% [46]. This fungus can break into rice cells and proliferate inside host cells, subsequently destroying the plant cells and leading to necrotic lesions. Rice blast disease can be divided into two types, leaf blast and neck blast, especially the latter, which can even cause empty panicles [47]. Planting rice cultivars with broad-spectrum disease resistance genes is a good strategy for preventing rice blast disease [48]. More than 30 *NLR* genes have been cloned in rice so far [49], including Piz-t [50], Pik [51], Pia [52], and Pib. Identifying resistance genes and the convergence of resistance genes in rice is the most effective and environmentally friendly approach for controlling rice blast disease [48]. Furthermore, the editing of susceptibility genes can enhance disease resistance in rice. For example, RESISTANCE TO BLAST1 (*RBL1*) negatively regulates disease resistance in rice. With the help of targeted genome editing, RBL1^Δ12^, an allele of RBL1, enhances pathogen resistance but does not decrease yield [48].

In rice, there are also six CAMTAs, but few reports have demonstrated the function of OsCAMTAs in disease resistance in rice. OsCBT (also known as OsCAMTA5) was reported to be a negative immune regulator, as the expression of various defense-related genes was increased in *oscbt-1* rice plants [53]. Transcriptional activation mediated by OsCBT can be inhibited by cotransfecting with CaM [54]. Our previous study showed that AtCAMTA3 negatively regulates plant immunity in Arabidopsis [32], but whether homologs of AtCAMTA3 contribute to resistance regulation in rice is unclear. CAMTAs play vital roles in the response to biotic stresses by modulating the expression of target genes. However, how CAMTAs confer immune function in rice to *M. oryzae* remains obscure. In this study, we found that there are three homologs, OsCAMTA1, OsCAMTA2, and OsCAMTA3, of AtCAMTA3 in rice. However, the *oscamta1/2* double mutant displayed wild-type-like phenotypes after infection with *M. oryzae*, suggesting that OsCAMTA1 and OsCAMTA2 may not regulate plant immunity. Then, *oscamta3* mutants were generated via the CRISPR/Cas9 gene editing system and the growth of *M. oryzae* was reduced in *oscamta3* mutants compared to that in wild-type ZH11, indicating that OsCAMTA3 negatively regulates disease resistance in rice. Through sequence alignment, we also identified a CAMTA protein that lacked the CG-1 domain and was named CAMTA PROTEIN LIKE (OsCAMTAPL). OsCAMTAPL was knocked out in ZH11 and the *oscamtapl* mutants also showed enhanced disease resistance to *M. oryzae*. Moreover, OsCAMTA3 interacted with OsCAMTAPL, and *oscamta3/pl* double mutants showed increased tiller numbers and enhanced resistance to *M. oryzae* compared with *oscamtapl* or *oscamta3* single mutants, suggesting that OsCAMTA3 may function together with OsCAMTAPL. To further investigate the immune function of OsCAMTA3 in rice, RNA-Seq analysis was performed between *oscamta3-1* and ZH11. Many genes, including *OsALDH2B1*, an acetaldehyde dehydrogenase that negatively regulates plant immunity [55], were significantly differentially expressed in *oscamta3-1* compared to ZH11. OsCAMTA3 could directly bind to the promoter of *OsALDH2B1*, and the expression of *OsALDH2B1* was significantly decreased in the *oscamta3*, *oscamtapl*, and *oscamta3/pl* mutants. Taken together, our results indicate that OsCAMTA3 and OsCAMTAPL can form a heterodimer to negatively regulate disease resistance in rice by activating the expression of *OsALDH2B1*.

## 2. Results

### 2.1. OsCAMTA3 Is a Negative Immune Regulator in Rice

**Figure 1:**
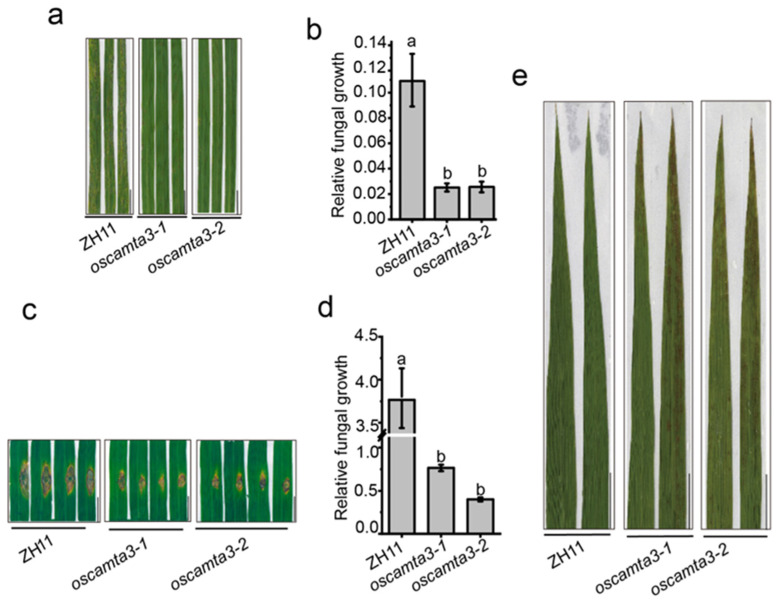
*Oscamta3* exhibited increased resistance to *M. oryzae*. (**a**) Fifteen-day-old ZH11 and *oscamta3* seedlings inoculated with *M. oryzae* by spraying. Images were taken at 5 days post inoculation (dpi). Bar = 1 cm. (**b**) Fungal biomass of spraying-inoculated leaves was measured to quantify relative fungal growth in ZH11 and *oscamta3*. Data are presented as the means ± SEs (*n* = 3). Lowercase letters indicate statistically significant differences (*p* < 0.05; one-way ANOVA). (**c**) Twenty-five-day-old ZH11 and *oscamta3* seedlings inoculated with *M. oryzae* by punch inoculation. Images were taken at 7 dpi. Bar = 1 cm. (**d**) Fungal biomass of punch-inoculated leaves was measured to quantify relative fungal growth in ZH11 and *oscamta3*. Data are presented as the means ± SEs (*n* = 3). Lowercase letters indicate statistically significant differences (*p* < 0.05; one-way ANOVA). (**e**) *Oscamta3* showed obvious lesion-mimic cell death at the heading stage. Bar = 1 cm.

To investigate the involvement of OsCAMTAs in disease resistance in rice, we performed protein sequence alignment to identify homologs of AtCAMTA3 in rice. As shown in Supplemental Figure S1 and Supplemental Table S1, OsCAMTA1 and OsCAMTA2 are the closest members to AtCAMTA3. Then, two independent null plants, *oscamta1/2-1* and *oscamta1/2-2*, were generated (Supplemental Figure S2). After challenging rice plants with *M. oryzae* by spraying, no differences in disease lesions were observed between *oscamta1/2* mutants and the wild type (Supplemental Figure S3a,b). This result was confirmed by performing punch inoculation assays and fungal biomass assays with the infected leaves of ZH11 and *oscamta1/2* mutants (Supplemental Figure S3c,d), suggesting that OsCAMTA1 and OsCAMTA2 may not be essential for disease resistance to *M. oryzae* in rice. Next, *OsCAMTA3*, another homolog of AtCAMTA3 (Supplemental Figure S1), was knocked out by CRISPR/Cas9, and two null mutants, *oscamta3-1* and *oscamta3-2*, were generated (Supplemental Figure S4). Then, ZH11, *oscamta3-1*, and *oscamta3-2* were inoculated with Zhong1 and *oscamta3-1* and *oscamta3-2* showed fewer disease lesions on their leaves than ZH11 (Figure 1a and Supplemental Figure S5). The biomass of relative fungal growth in *oscamta3* was significantly lower than that in ZH11 (Figure 1b). The phenotype of *oscamta3* mutants were also examined by the punch method and similar results were obtained (Figure 1c,d), indicating that *oscamta3* mutants displayed enhanced disease resistance to *M. oryzae*. To further investigate the immune function of OsCAMTA3 in rice, the expression of *OsCAMTA3* and *PR* genes, including *OsOPR1*, *OsPR5*, *OsPR8*, *OsPR10*, and *OsWRKY45*, was examined after inoculation with *M. oryzae*. The expression of *OsCAMTA3* was not induced after *M. oryzae* infection, but the expression of *PR* genes was significantly increased in *oscamta3* mutants compared to ZH11 after inoculation with *M. oryzae* (Supplemental Figure S6). Additionally, *oscamta3-1* and *oscamta3-2* showed lesion-mimic cell death at the heading stage (Figure 1e), indicating that *oscamta3* mutants exhibit autoimmunity. Taken together, these results indicate that *OsCAMTA3* negatively regulates disease resistance in rice and that loss of function of OsCAMTA3 leads to enhanced disease resistance.

### 2.2. OsCAMTAPL Negatively Regulates Disease Resistance in Rice

**Figure 2:**
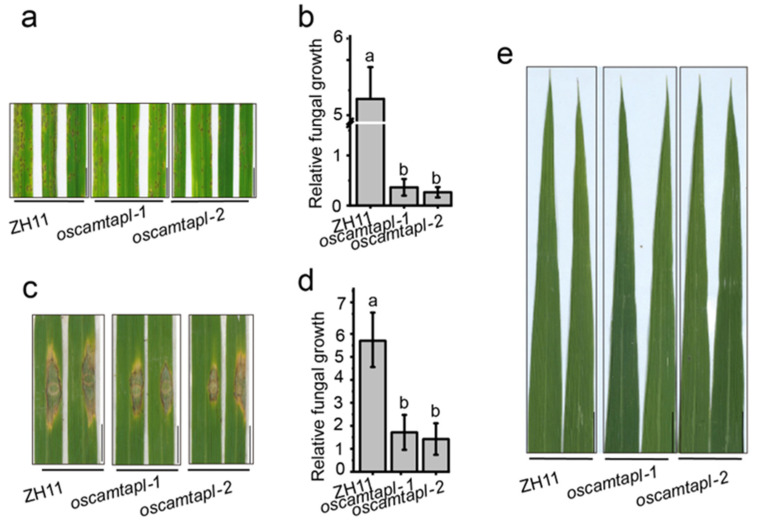
*Oscamtapl* exhibited increased resistance to *M. oryzae*. (**a**) Fifteen-day-old ZH11 and *oscamtapl* seedlings inoculated with *M. oryzae* Zhong1 by spraying. Images were taken at 5 dpi. Bar = 1 cm. (**b**) Fungal biomass of spraying-inoculated leaves was measured to quantify relative fungal growth in ZH11 and *oscamtapl*. Data are presented as the means ± SEs (*n* = 3). Lowercase letters indicate statistically significant differences (*p* < 0.05; one-way ANOVA). (**c**) Twenty-five-day-old ZH11 and *oscamtapl* seedlings inoculated with *M. oryzae* Zhong1 by punch inoculation. Images were taken at 7 dpi. Bar = 1 cm. (**d**) Fungal biomass of punch-inoculated leaves was measured to quantify relative fungal growth in ZH11 and *oscamtapl*. Data are presented as the means ± SEs (*n* = 3). Lowercase letters indicate statistically significant differences (*p* < 0.05; one-way ANOVA). (**e**) *Oscamtapl* did not show an obvious hypersensitive response at the heading stage. Bar = 1 cm.

Through sequence alignment, a special CAMTA protein, OsCAMTAPL, which was highly homologous to OsCAMTA3, but lacked the CG-1 domain, was found (Supplemental Figure S1b). The expression of *OsCAMTAPL* was not induced by *M. oryzae* (Supplemental Figure S7a), which was similar to *OsCAMTA3* (Supplemental Figure S6a). To analyze the immune function of OsCAMTAPL in rice, two null mutants, *oscamtapl-1* and *oscamtapl-2*, were generated (Supplemental Figure S8). After inoculation with Zhong1, both *oscamtapl-1* and *oscamtapl-2* showed enhanced resistance to *M. oryzae* (Figure 2a–d and Supplemental Figure S9). Moreover, the transcript levels of *OsPR1* and *OsWRKY45* were significantly increased in *oscamtapl* mutants compared with ZH11 after inoculation with *M. oryzae* (Supplemental Figure S7b), indicating that OsCAMTAPL was a negative regulator of disease resistance against *M. oryzae* in rice. However, *oscamtapl* did not exhibit spontaneous cell death at the heading stage (Figure 2e), which was different from the *oscamta3* mutants.

### 2.3. OsCAMTA3 and OsCAMTAPL Function Together to Negatively Regulate Disease Resistance in Rice

**Figure 3:**
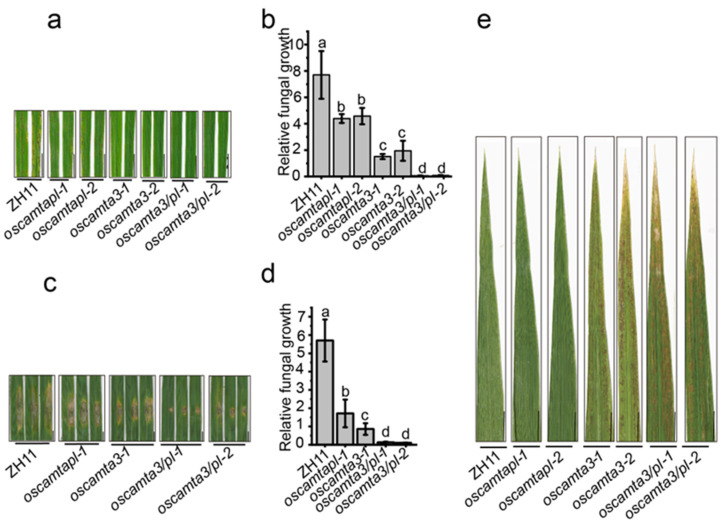
*Oscamta3/pl* exhibited increased resistance to *M. oryzae*. (**a**) Fifteen-day-old ZH11, *oscamta3*, *oscamtapl*, and *oscamta3/pl* seedlings inoculated with *M. oryzae* by spraying. Images were taken at 5 dpi. Bar = 1 cm. (**b**) Fungal biomass of spraying-inoculated leaves was measured to quantify relative fungal growth in ZH11, *oscamta3*, *oscamtapl*, and *oscamta3/pl*. Data are presented as the means ± SEs (*n* = 3). Lowercase letters indicate statistically significant differences (*p* < 0.05; one-way ANOVA). (**c**) Twenty-five-day-old ZH11, *oscamta3*, *oscamtapl*, and *oscamta3/pl* seedlings inoculated with *M. oryzae* by punch inoculation. Images were taken at 7 dpi. (**d**) Fungal biomass of punch-inoculated leaves was measured to quantify relative fungal growth in ZH11 and *oscamta3/pl*. Data are presented as the means ± SEs (*n* = 3). Lowercase letters indicate statistically significant differences (*p* < 0.05; one-way ANOVA). (**e**) *Oscamta3/pl* plants showed obvious lesion-mimic cell death at the heading stage. Bar = 1 cm.

As both *oscamta3* and *oscamtapl* showed enhanced disease resistance to *M. oryzae*, *OsCAMTA3* and *OsCAMTAPL* were knocked out simultaneously to generate independent *oscamta3/pl* lines (Supplemental Figure S10). With *oscamta3* and *oscamtapl* mutants as controls, the *oscamta3/pl-1* and *oscamta3/pl-2* mutants displayed an approximately 100-fold increase in disease resistance compared with *oscamta3* or *oscamtapl* after inoculation with Zhong1 (Figure 3a–d and Supplemental Figure S11), indicating that *OsCAMTA3* and *OsCAMTAPL* showed important functions in disease resistance in rice. Moreover, the *oscamta3/pl* double mutant showed obvious cell death at the heading stage (Figure 3e). Then, the expression of *OsPR5*, *OsPR10*, and *OsWRKY45* were detected in ZH11, *oscamta3-1*, *oscamtapl-1*, and *oscamta3/pl-1* and the expression of *PR* genes in *oscamta3/pl-1* was much higher than that in ZH11, *oscamta3-1*, and *oscamtapl-1* (Supplemental Figure S12), indicating that OsCAMTA3 and OsCAMTAPL play a crucial role in disease resistance in rice.

### 2.4. OsCAMTA3 Associates with OsCAMTAPL

**Figure 4:**
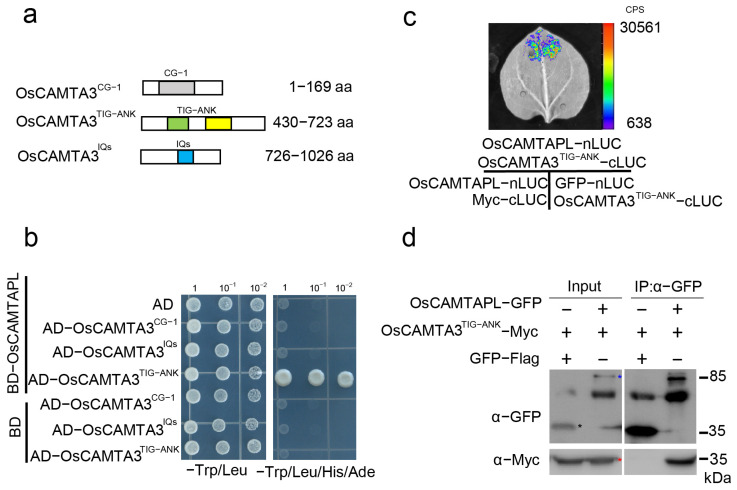
OsCAMTAPL interacted with the TIG-ANK domain of OsCAMTA3. (**a**) The different domains of OsCAMTA3 are shown. OsCAMTA3 contains the CG-1 (the gray area), TIG-ANK(the cyan-yellow area) and IQ (the blue area) domains. The numbers indicate the positions of amino acid sequences. (**b**) The TIG-ANK domain of OsCAMTA3 interacted with OsCAMTAPL in a Y2H assay. The coding sequence (CDS) of *OsCAMTA3^CG−1^*, *OsCAMTA3^TIG-ANK^*, or *OsCAMTA3^IQs^* was fused to the vector pGADT7 (AD), and the CDS of *OsCAMTAPL* was fused to pGBKT7 (BD). The empty vectors pGADT7 (AD) and pGBKT7 (BD) were used as negative controls. Yeast cells containing BD-OsCAMTAPL and AD-OsCAMTA3^TIG-ANK^ plasmids were grown on SD-Leu-Trp-His-Ade medium. (**c**) Images of *N. benthamiana* leaves with the indicated constructs in LUC assays. The coding sequence (CDS) of *OsCAMTA3^TIG-ANK^* was fused to the C-terminal fragment of firefly luciferase (cLUC), and the CDS of *OsCAMTAPL* was fused to the N-terminal fragment of firefly luciferase (nLUC). Green fluorescence protein (GFP)-nLUC and Myc-nLUC were used as negative controls. The indicated constructs were transiently coexpressed in 4-week-old *N. benthamiana* leaves, and the bioluminescence images were captured by a CCD camera. (**d**) Co-IP assay was performed by transiently coexpressing OsCAMTA3^TIG-ANK^-Myc and OsCAMTAPL-Flag in *N. benthamiana* leaves. Total protein was extracted and subjected to immunoprecipitation by anti-Flag beads. Immunoblotting analysis was performed with anti-Myc and anti-Flag antibodies. OsCAMTAPL-Flag, GFP-Flag, and OsCAMTA3^TIG-ANK^-Myc are indicated by blue, black, and red asterisks. Full-length blots are presented in Supplementary Figure S6.

As OsCAMTAPL lacks the CG-1 domain, we proposed that OsCAMTAPL may act as a transcription cofactor that modulates the activation of other TFs. In this case, OsCAMTAPL may interact with OsCAMTA3. OsCAMTA3 mainly contains three domains: the N-terminal CG-1 domain, TIG-ANK domain, and C-terminal IQs domain. To analyze which domain of OsCAMTA3 is responsible for the potential interaction with OsCAMTAPL, several truncated forms of OsCAMTA3 were generated (Figure 4a) and yeast two-hybrid (Y2H) assays were performed. As shown in Figure 4b, only AD-OsCAMTA3^TIG-ANK^ interacted with OsCAMTAPL but not with AD-OsCAMTA3^CG−1^ or AD-OsCAMTA3^IQs^, indicating that OsCAMTAPL interacted with the TIG-ANK domain of OsCAMTA3. To confirm this association, we performed split-luciferase complementation (LUC) assays and found that a strong luminescence signal was observed in *Nicotiana benthamiana* leaves coexpressing OsCAMTAPL–nLUC with OsCAMTA3^TIG-ANK^–cLUC (Figure 4c). Next, coimmunoprecipitation (co-IP) assays were performed by coexpressing OsCAMTAPL–Flag and OsCAMTA3^TIG-ANK^–Myc in *N. benthamiana* leaves. The co-IP assay was carried out with an anti-Flag antibody and subsequently detected by immunoblotting with anti-Myc and anti-Flag antibodies. As shown in Figure 4d and Supplemental Figure S13, OsCAMTA3^TIG-ANK^ interacted with OsCAMTAPL in *N. benthamiana* leaves. Together, these results indicate that OsCAMTA3 can associate with OsCAMTAPL via its TIG-ANK domain.

### 2.5. OsCAMTA3 and OsCAMTAPL Contribute to Plant Architecture

**Figure 5:**
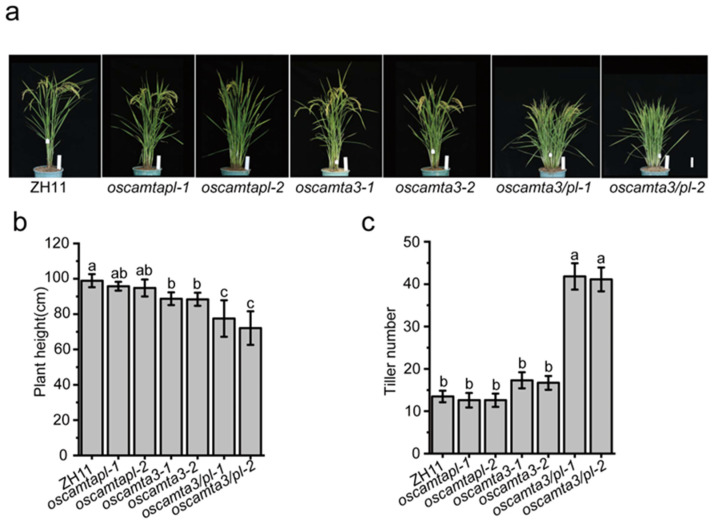
Morphology of ZH11 and *oscamtas* plants at the heading stage. (**a**) Phenotypes of ZH11 and *oscamtas* plants at the heading stage. Bars = 10 cm. (**b**,**c**) Comparison of plant height and tiller number between ZH11 and *oscamtas* plants. Data are presented as the means ± SE (*n* = 10). Lowercase letters indicate statistically significant differences (*p* < 0.05; one-way ANOVA).

Enhanced immunity is often accompanied by impaired growth and yield. Consistently, at the heading stage, the plant height of *oscamtapl* plants did not appear significantly different compared to that of ZH11, but the plant height of *oscamta3* and *oscamta3/pl* was significantly reduced compared to that of ZH11 (Figure 5a,b). Moreover, the tiller number of *oscamta3/pl*, but not of *oscamta3* and *oscamtapl*, was significantly increased compared with that of ZH11 (Figure 5c), indicating that knocking out *OsCAMTA3* and *OsCAMTAPL* affects rice growth and development.

### 2.6. Many Genes Are Differentially Expressed between ZH11 and oscamta3-1

**Figure 6:**
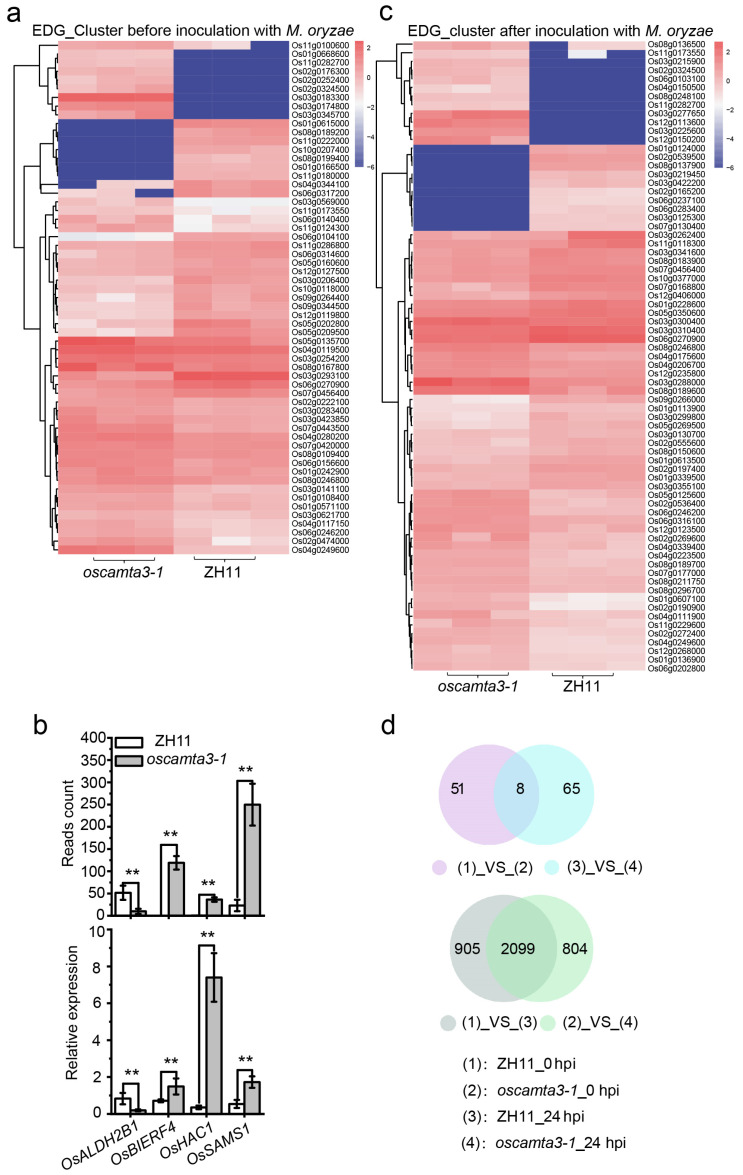
Identification of DEGs in ZH11 and *oscamta3-1* by RNA-seq. (**a**) The number of DEGs between ZH11 and *oscamta3-1* before inoculation with *M. oryzae*. (**b**) *OsALDH2B1*, *OsBIERF1*, *OsHAC1*, and *OsSAMS1* were chosen to verify the RNA-Seq results by qRT-PCR. Data are presented as the means ± SEs (*n* = 3). (**; *p* < 0.01; Student’s *t*-test). (**c**) The number of DEGs between ZH11 and *oscamta3-1* after inoculation with *M. oryzae*. (**d**) Venn diagrams showing the number of EDGs before or after inoculation with *M. oryzae*.

To determine the immune function of OsCAMTA3 in rice, a transcriptome deep sequence (RNA-seq) analysis of ZH11 and *oscamta3-1* leaves was performed before and after inoculation with *M. oryzae* strain Guy11. The *oscamta3-1* materials showed increased disease resistance compared with ZH11 (Supplemental Figure S14). A pairwise comparison was conducted to identify differentially expressed genes (DEGs; genes, FDR < 0.01, |log_2_ (fold change)| > 0) between ZH11 and *oscamta3-1* before inoculation with Guy11 and detected 59 genes significantly differentially expressed in *oscamta3-1* relative to ZH11, consisting of 23 upregulated and 36 downregulated DEGs (Figure 6a). The expression of four genes, namely, OsALDH2B1 [55], *OsBIERF4* [56], *OsHAC1* [57], and *OsSAMS1* [58], was verified via qRT-PCR analysis in the RNA-seq dataset. As shown in Figure 6b, the expression of these genes was similar to the RNA-seq results.

Additionally, 73 DEGs were identified between ZH11 and *oscamta3-1* after inoculation with Guy11 (Figure 6c). Moreover, as shown in Figure 6d, the expression of eight genes was consistently changed in ZH11 and *oscamta3-1* (Figure 6d and Supplemental Table S2). In total, 3004 and 2903 EDGs were identified in ZH11 and *oscamta3-1*, respectively, in the 24 h time-series of the fungal infection (Supplemental Table S3). Among these DEGs, 905 and 804 were specifically regulated in ZH11 and *oscamta3-1*, respectively (Figure 6d), suggesting that these genes may be regulated by OsCAMTA3, and the enhanced disease resistance in *oscamta3-1* may be due to the dramatic transcriptional changes in these genes.

**Figure 7:**
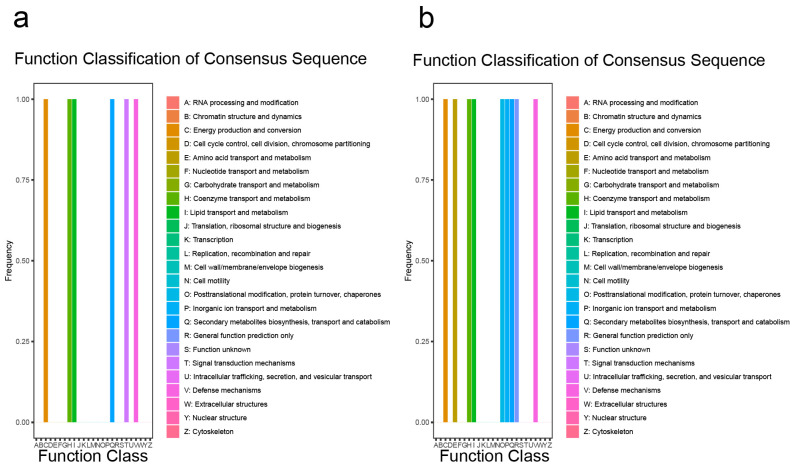
Identification of the function of DEGs in ZH11 and *oscamta3-1*. (**a**) Functional classification of consensus sequence term enrichment analysis of all DEGs before inoculation with *M. oryzae*. (**b**) Functional classification of consensus sequence term enrichment analysis of all DEGs after inoculation with *M. oryzae*.

Functional classification of the consensus sequence indicated that the DEGs before inoculation with *M. oryzae* were enriched in the transport and metabolism of amino acids, coenzymes, lipids, inorganic ions, secondary metabolite biosynthesis, or defense mechanisms (Figure 7a). Functional classification of the consensus sequence indicated that the DEGs after inoculation with *M. oryzae* were similar to the DEGs before inoculation with *M. oryzae* (Figure 7b). An analysis of Kyoto Encyclopedia of Genes and Genomes (KEGG) terms revealed that genes among DEGs before inoculation with *M. oryzae* were enriched in the pathway of secondary metabolites, for example, the degradation or metabolism of fatty acids, valine, leucine, lysine, cysteine, and methionine (Supplemental Figure S15a); the KEGG terms revealed that genes among DEGs after inoculation with *M. oryzae* were similar to the DEGs before inoculation with *M. oryzae* (Supplemental Figure S15b). All of these results suggest that OsCAMTA3 may play an important role in different pathways.

### 2.7. OsCAMTA3 Binds to the Promoter of OsALDH2B1 to Regulate Its Expression

**Figure 8:**
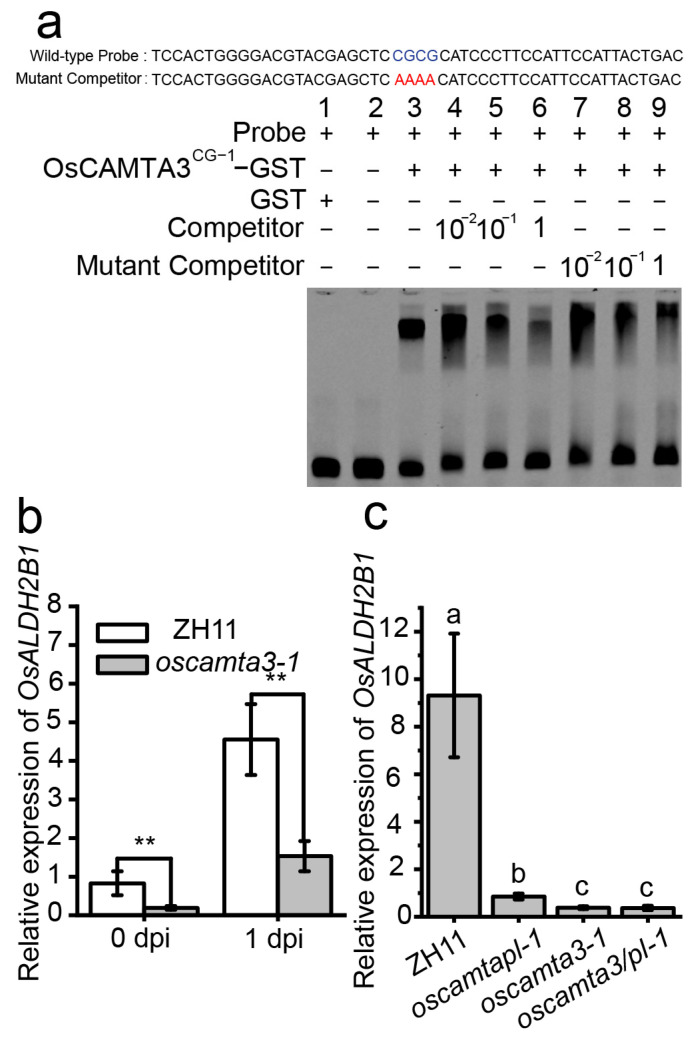
OsCAMTA3 activated the expression of OsALDH2B1 by binding to its CGCG motif. (**a**) OsCAMTA3^CG−1^ bound to the DNA fragment of the *OsALDH2B1* promoter containing the CGCG motif in an EMSA. Sequences of the probe and mutated probe are presented at the top. The wild-type CGCG motif and the mutant AAAA motif have been indicated by blue and red, respectively. Full-length blots are presented in Supplementary Figure S9. (**b**) qRT-PCR analysis of *OsALDH2B1* expression in ZH11 and *oscamta3-1* before and after inoculation with *M. oryzae*. Data are presented as the means ± SEs (*n* = 3). (**; *p* < 0.01; Student’s *t*-test). (**c**) The transcripts of *OsALDH2B1* in ZH11, *oscamta1/2-1*, *oscamta3-1*, *oscamtapl-1*, and *oscamta3/pl-1* mutants. Data are presented as the means ± SEs (*n* = 3). Lowercase letters represent statistically significant differences (*p* < 0.05; one-way ANOVA).

*OsALDH2B1*, one of the EDGs between ZH11 and *oscamta3*, has been reported to be an aldehyde dehydrogenase (ALDH) TF, and the loss of function of *OsALDH2B1* results in increased resistance to many pathogens [55]. We observed a 5′-CGCG-3′ (117–120 bp) motif, which can be targeted by OsCAMTA3, upstream of the transcription start site of the CDS of *OsALDH2B1* (Figure 8a). Therefore, we speculated that *OsALDH2B1* may be a candidate target gene of OsCAMTA3. To test this hypothesis, an electrophoretic mobility shift assay (EMSA) was performed. As the CG-1 DNA-binding domain of OsCAMTA3 determines its ability to bind the CGCG motif, the CAMTA3^CG−1^-GST protein was purified. The GST protein was also purified for use as a negative control. In this EMSA, one CGCG motif-containing sequence from the *OsALDH2B1* promoter was labeled by Cy5 as a probe, and the no-Cy5-labeled sequence was used as a competitor. A mutant competitor whose CGCG motif was mutated to AAAA was also prepared (Figure 8a), and OsCAMTA3^CG−1^-GST, but not GST, attached to the Cy5-labeled *OsALDH2B1* probe (Figure 8a). To confirm this result, we added a competitor and found that the binding of the Cy5-labeled *OsALDH2B1* probe by OsCAMTA3^CG−1^-GST was reduced (Figure 8a and Supplemental Figure S16), indicating that OsCAMTA3 could bind to the promoter of *OsALDH2B1* directly.

Then, the expression of *OsALDH2B1* after inoculation with *M. oryzae* was investigated by qRT-PCR analysis. The expression of *OsALDH2B1* was reduced in *oscamta3* compared to ZH11 (Figure 8b), indicating that OsCAMTA3 may regulate the expression of *OsALDH2B1*. Next, the expression of *OsALDH2B1* was detected in ZH11, *oscamta3-1*, *oscamtapl-1*, and *oscamta3/pl-1*. The expression of *OsALDH2B1* was reduced in *oscamta3-1*, *oscamtapl-1*, and *oscamta3/pl-1* (Figure 8c), suggesting that OsCAMTA3 and OsCAMTAPL may negatively regulate disease resistance by activating the expression of *OsALDH2B1* in rice.

## 3. Discussion

### 3.1. OsCAMTA3 and OsCAMTAPL Negatively Regulate Disease Resistance in Rice

Although the function of AtCAMTA in Arabidopsis has been well characterized, whether OsCAMTAs contribute to the regulation of disease resistance in rice is unclear. In this study, we found that OsCAMTA3 acted as a negative regulator in disease resistance in rice. OsCAMTAPL, a homolog of OsCAMTA3 lacking the DNA binding domain, interacted with OsCAMTA3. The loss of function of OsCAMTA3 or OsCAMTAPL led to enhanced disease resistance to *M. oryzae*. Moreover, *oscamta3/pl* double mutants displayed more robust disease resistance to *M. oryzae* than the *oscamta3* or *oscamtapl* single mutant. The RNA-seq analysis showed that the expression of *OsALDH2B1* was reduced in *oscamta3-1*. The CG-1 domain of OsCAMTA3 binds to the promoter of *OsALDH2B1* to regulate its expression, indicating that the enhanced disease resistance in *oscamta3*, *oscamtapl*, and *oscamta3*/*pl* may be the result of the downregulation of *OsALDH2B1*.

### 3.2. Trade-Off Regulation of Rice Growth and Development by OsCAMTA3

Previous studies have indicated that TFs play an essential role in balancing plant growth and immunity. For example, IDEAL PLANT ARCHITECTURE 1 (IPA1), a SQUAMOSA promoter binding protein-like (SPL) TF, plays an important role in promoting yield and disease resistance in rice [59]. The tiller number of *oscamta3/pl* double mutants was significantly increased compared with that of ZH11, suggesting that OsCAMTA3 may also play a role in rice development by regulating the expression of *OsALDH2B1*. OsALDH2B1 has been reported to be a TF that regulates the BR signaling pathway [55]. Therefore, the increased tiller number may result from the overdose of BR or overactivated BR signaling in rice because OsALDH2B1 can interact with and inhibit the expression of OsBZR1, a key component of the BR signaling pathway determining the number of rice tillers and positively regulating disease resistance in rice [55,60]. We also detected that the expression of *OsBZR1* in *oscamta3/pl* was significantly increased compared with that in ZH11 (Supplemental Figure S17), which was consistent with the downregulation of *OsALDH2B1* expression in *oscamta3/pl* mutants. Because OsCAMTA3 regulates the expression of *OsALDH2B1*, the loss of function of OsCAMTA3/PL may lead to the imbalanced expression of *OsALDH2B1* and *OsBZR1*, resulting in robust disease resistance to *M. oryzae* and an increased tiller number in *oscamta3/pl* mutants. This result is consistent with a previous finding that the ubiquitin-conjugating enzyme (UBC) member UBC2 negatively regulates plant immunity by interacting with and suppressing the expression of SGT1 [61]. Thus, further investigations are needed to analyze whether OsBZR1 contributes to the enhanced resistance and tiller number in *oscamta3/pl* mutants.

### 3.3. Regulation of CAMTAs in Plants

The negative immune regulators AtCAMTA1, AtCAMTA2, and AtCAMTA3 show redundant functions in repressing the SA pathway [33]. AtCAMTA3 has been reported to be a negative TF in plant immunity [25]. However, in our study, we found that the *oscamta1/2* double mutant did not affect disease resistance, suggesting that OsCAMTA1 and OsCAMTA2 may not contribute to disease resistance in rice and that there may be different functional mechanisms between Arabidopsis and rice [62]. It is also interesting to knock out *OsCAMTA3* in the background of *oscamta1/2* to generate *oscamta1/2/3* triple mutants and to examine their phenotypes with *oscamta1/2* and *oscamta3* mutants as controls. Furthermore, OsCAMTAPL lacks the CG-1 domain, which is required for binding to the DNA sequence, but the expression of *OsALDH2B1* was also significantly decreased in the *oscamtapl* single mutant. As OsCAMTAPL interacts with the TIG-ANK domain behind the CG-1 domain of OsCAMTA3, the most likely reason for this observation is that OsCAMTAPL may associate with OsCAMTA3 and stabilize its DNA binding ability, which remains to be addressed in future studies.

How OsCAMTA3/PL receives the immune signal remains obscure. Since OsCaM can interact with and inhibit the transcriptional activity of OsCBT [53], we speculate that the transcriptional activity of OsCAMTA3/PL may also be suppressed by OsCaMs or OsCMLs after the perception of pathogens. In the resting state, OsCAMTA3/PL binds to their target genes and repress immunity. Upon pathogen infection, the influxed Ca^2+^ is sensed by OsCaMs or OsCMLs, leading to the release of OsCAMTA3/PL and the activation of resistance. Moreover, in Arabidopsis, AtCPK5/6 have been reported to be Ca^2+^ sensors and regulate camalexin biosynthesis by interacting with and phosphorylating the TF AtWRKY33, which plays an important role in plant immunity [63]. AtCPK5 also phosphorylates AtCAMTA3 directly to increase its destabilization [64]. CBL, another Ca^2+^ sensor in plants, can interact with the CBL-interacting protein kinase (CIPK) and form a CBL–CIPK complex to transmit the signal downstream [23,65]. For instance, the OsCBL8–OsCIPK17 complex plays a negative role in seedling growth and development [66]. Therefore, OsCAMTA3/PL may also be regulated by some protein kinases, such as OsCPKs or OsCIPKs, after recognition of pathogens in rice. Interestingly, OsCAMTAPL, the truncated CAMTA protein, is not present in the model plant Arabidopsis. The association of OsCAMTA3 and a truncated OsCAMTA protein to regulate downstream gene expression may represent a new mechanism deployed by CAMTAs in plants.

### 3.4. The Potential Application of OsCAMTA3 and OsCAMTAPL in Breeding

The function of CAMTAs in disease resistance in rice has not been reported. Our work uncovers the immune function of OsCAMTA3 and OsCAMTAPL in rice, which is highly important for agricultural production. With the development of genome editing discoveries and applications, whether OsCAMTA3 and OsCAMTAPL can be applied in breeding for improving disease resistance in rice needs to be explored in the future. Moreover, the application of DNA markers for screening *oscamta3* and *oscamtapl* breeding materials will make it much easier and faster to screen resistant materials.

## 4. Materials and Methods

### 4.1. Plant Materials and Growth Conditions [67]

The japonica rice (*Oryza sativa* L.) plants used in this study included wild-type ZH11 and the mutants *oscamta1/2-1*, *oscamta1/2-2*, *oscamta3-1*, *oscamta3-2*, *oscamtapl-1*, *oscamtapl-2*, *oscamta3/pl-1*, and *oscamta3/pl-2*. All mutants were generated through the CRISPR/Cas9 gene editing system in the ZH11 background. Briefly, the guide SG/gRNA sequence of the target gene was cloned and inserted into the CRISPR/Cas9 gene editing vector BGK03. The recombinant tRNA-gRNA-CRISPR/Cas9 vector was subsequently introduced into rice embryonic callus via *Agrobacterium tumefaciens*. Hygromycin resistance MS medium was used to screen for positive plants. The target sequence of the indicated gene was amplified via PCR and sequenced. The primers used for cloning the coding sequences by PCR can be found in Supplemental Table S4. All rice plants were grown in greenhouses at 28 °C with a 12 h light/12 h dark cycle and 65% humidity or in paddy fields in FUJIAN, China.

### 4.2. Inoculation of Blast Fungus and Fungal Biomass Assays [67]

The rice blast fungus strain Zhong1 was cultured on CM medium and then grown on rice bran medium for 10 days in the dark at 28 °C. After the aerial hyphae were flattened off, the plates were incubated under light (12 h light/12 h dark cycle, 28 °C) for sporulation. The conidial spores were collected in water with 0.02% (*v*/*v*) Tween-20. Spraying was carried out with 15-day-old seedlings, and the sprayed seedlings were placed into sealed containers to maintain humidity and grown in the dark for 24 h. Then, the inoculated plants were returned to the greenhouses. The appropriate amount of water was sprayed on the rice leaves to maintain humidity every 12 h. The diseased leaves were photographed 6–7 days after inoculation, and three sprayed leaves were collected for the relative fungal biomass assay. The use of punch inoculation was more accurate than sprayed inoculation systems. Punch inoculation, another system of making some of the small disease resistance phenotypes more obvious, was carried out with 25-day-old seedlings. In brief, rice leaves were performed on press-injured spots of 2.0 mm diameter made with a mouse ear punch, and the spore suspension (10 µL) was placed on the injured spots with Scotch tape. The inoculated plants were returned to the greenhouses. The appropriate amount of water was sprayed on the rice leaves to maintain humidity every 12 h. The injured lesion was cut for imaging 8–10 days after inoculation, and the Adobe Photoshop 2022 software package was used to measure the lesion area. Then, three punched leaves were collected for the relative fungal biomass assay. For fungal biomass assays, three punched or sprayed leaves detached from different plants were collected for DNA extraction via the standard Cetyltrimethyl ammonium bromide extraction protocol, and DNA-based qPCR was used to measure relative fungal growth. The threshold cycle value of the *M. oryzae Pot2* DNA against the threshold cycle of the rice genomic ubiquitin DNA was obtained. The sequences of primers used for determining relative fungal growth via DNA-based qPCR can be found in Supplemental Table S4.

### 4.3. qRT-PCR Analysis [68]

The leaves of 15-day-old rice seedlings sprayed with conidial spores for 0, 1, or 2 days were collected to determine the relative expression of indicated genes. Three seedlings were collected at each time point as one biological replicate, and all RT-PCR analyses were performed on three biological replicates. Total RNA was extracted from leaf tissues using TRIzol reagent as described previously, and 2 µg of RNA was subjected to first-strand cDNA synthesis using EasyScript One-Step gDNA Removal and cDNA Synthesis SuperMix. qRT-PCR was then performed using PerfectStart Green qPCR SuperMix with a CFX Connect Real-time system. Sequences of the primers used for amplifying the plant defense-related genes are provided in Supplemental Table S4.

### 4.4. Y2H Assay [69]

The coding sequence of *OsCAMTAPL* was cloned and inserted into a pGBKT7 vector. The coding sequence of *OsCAMTA3* was divided, cloned, and inserted into the bait vector pGADT7. The appropriate pairs of constructs were transformed into yeast strain AH109. The yeast clones were grown on synthetic defined (SD) medium lacking Trp and Leu (SD-Trp-Leu) at 30 °C for 2 days and then spotted onto SD medium lacking Trp, Leu, His, and Ade (SD-Trp-Leu-His-Ade) to detect interactions.

### 4.5. Firefly Split-LUC Assay [69]

The coding sequences of *OsCAMTAPL* and the truncated fragments of *OsCAMTA3* were cloned and inserted into the nLUC or cLUC vector, respectively, as described previously. Different construct pairs were then transiently coexpressed in *N. benthamiana* leaves via *Agrobacterium tumefaciens* strain GV3101 with agrobacteria resuspended (OD_600_ = 0.6) in infiltration buffer (10 mM MgCl_2_, 10 mM MES, 200 mM acetosyringone, pH 5.6). Two days post infiltration, 1 mM D-luciferin was sprayed onto detached leaves, which were then kept in the dark for 5 min. LUC activity was detected using the CCD plant imaging system.

### 4.6. Co-IP Assay [70]

Co-IP assays were performed in *N. benthamiana* as described previously. Total protein was extracted after Agrobacterium-mediated infiltration for 2 days with extraction buffer (50 mM Tris-HCl pH 7.5, 150 mM NaCl, 1 mM EDTA, 1 mM DTT, 1% [*v*/*v*] IGEPAL CA-630, 10% [*v*/*v*] glycerol, 1 mM PMSF, and protease inhibitor cocktail). For immunoprecipitation with an anti-Flag antibody, total protein was incubated with 10 µL of Myc nanobeads at 4 °C for 2 h with gentle shaking. After incubation, the beads were washed three times with wash buffer (50 mM Tris-HCl at pH 7.5, 150 mM NaCl, 1 mM EDTA, 1 mM DTT, 1 mM PMSF, 10% glycerol, 0.3% [*v*/*v*] IGEPAL CA-630) and boiled at 98 °C for 10 min. The samples were separated by SDS-PAGE (10%) and analyzed by immunoblotting using anti-Flag and anti-Myc antibodies.

### 4.7. RNA-Sequencing Analysis [71]

Fifteen-day-old rice plants, including ZH11 and *oscamta3-1*, were prepared under normal conditions and collected before and after inoculation with Guy11 for 24 h. Three rice plants as one biological replicate, and the RNA-sequencing analysis was performed on three biological replicates. Sample extraction and Illumina sequencing were performed by Berry Genomics (https://www.berrygenom-ics.com/, accessed on 30 April 2024). Clean reads were mapped to the rice genome (MSU-RGAP 7.0) using HISAT2, and Pearson’s correlation coefficient was used to judge the biological replicates. The DEGs were identified by pairwise comparison (FDR < 0.01, |log_2_ (fold change)| > 0). The final clustering allowed us a quick look at the DEG expression levels and statistical significance between ZH11 and *oscamta3-1*.

### 4.8. Recombinant Protein Expression [64]

The coding sequence of the OsCAMTA3^CG−1^ fragment was cloned and inserted into the vector pGEX4T-1 and expressed in *E. coli* strain BL21 (DE3) under 0.4 mM IPTG at 16 °C for 24~28 h. The cell lysis extraction was purified by amylose resin. Relevant primer sequences and constructs are given in Supplementary Table S4.

### 4.9. Electrophoretic Mobility Shift Assay (EMSA) [62]

A 50 bp DNA fragment of the *OsALDH2B1* promoter containing the CGCG motif was synthesized with a Cy5 end label as a probe, and the competing probe was synthesized without a Cy5 label. The probes were incubated with the indicated amount of recombinant GST-tagged protein in a 20 lL reaction (100 mM Tris-HCl pH 7.5, 100 mM KCl, 50 mM MgCl_2_, 1 mM DTT, 0.05 mg/mL poly [dI-dC]) at 4 °C for 30 min. For commotion assays, 1-, 10-, or 100-fold unlabeled competitor DNA was added to the reaction before the addition of the Cy5-labeled probes. The polyacrylamide gel containing 3.5% (*w*/*v*) acrylamide was prerun in 0.5 × Tris borate EDTA running buffer for 1 h at 4 °C in the dark. Then, the protein–DNA mixtures were added and electrophoresed for 1 h at 4 °C with a voltage of 100 V. Gels were directly scanned using an Odyssey CLx Infrared Imaging System.

## 5. Conclusions

Our results indicate that OsCAMTA3 plays a crucial role in disease resistance in rice. OsCAMTA3 and OsCAMTAPL can form a heterodimer and positively regulate the expression of *OsALDH2B1* in rice. Knock-out OsCAMTA3 or OsCAMTAPL led to enhanced disease resistance to rice blast disease. OsCAMTA3 could bind to the promoter of *OsALDH2B1* and regulate its expression. Consistently, the expression of *OsALDH2B1* was reduced in *oscamta3*, *oscamtapl*, and *oscamta3*/*pl*. The reduced expression of *OsALDH2B1* in *oscamta3/pl* mutants may lead to the high expression of *OsBZR1*, resulting in robust resistance to *M. oryzae* and an increased tiller number in *oscamta3/pl*, which is useful for crop disease resistance breeding.

## Data Availability

Sequence data of proteins (Supplemental Figure S1) in this study can be found at https://www.arabidopsis.org/browse/Cereon/index.jsp (accessed on 1 May 2023). and https://www.ncbi.nlm.nih.gov/ (accessed on 1 May 2023). Sequence information from this article can be found in the annotation project database under the following accession numbers: *OsCAMTA1* (*Os03g0191000*), *OsCAMTA2* (*Os10g0375600*), *OsCAMTA3* (*Os07g0623100*), *OsCAMTAPL* (*Os03g0388500*), *OsCAMTA4* (*Os01g0923600*), *OsCAMTA5* (*Os07g0490200*), *OsCAMTA6* (*Os04g0388500*), *OsALDH2B1* (*Os06g0270900*), *OsBIERF4* (*Os03g0183300*), *OsSAMS1* (*Os05g0135700*), *OsOPR1* (*Os06g0216300*), *OsPR5* (*Os12g0628600*), *OsPR8* (*Os10g0416500*), *OsPR10* (*Os12g0555500*), *OsWRKY45* (*Os05g0322900*), *OsBZR1* (*Os07g0580500*), and *Ubiqitin* (*Os03g0234200*).

## Supplementary Materials

The following supporting information can be downloaded at: https://www.mdpi.com/article/10.3390/ijms25095049/s1.

## Abbreviations

ZH11: Zhonghua11; CAMTA: calmodulin-binding transcriptional activator; TF: transcription factor; PRRs: pattern recognition receptors; PAMPs or DAMPs: pathogen-/damage-associated patterns; PTI: pattern-triggered immunity; NLRs: nucleotide-binding domain leucine-rich-repeat-containing receptors; ETI: effector-triggered immunity; Ca^2+^: calcium ions; ROS: reactive oxygen species; PR: pathogenesis-related; CaM: calmodulin; CPK: calcium-dependent protein kinase; SA: salicylic acid; *M. oryzae*: *Magnaporthe oryzae*; Y2H: two-hybrid; LUC: luciferase complementation; co-IP: coimmunoprecipitation; *N. benthamiana*: *Nicotiana benthamiana*; DEGs: differentially expressed genes; KEGG: Kyoto Encyclopedia of Genes and Genomes; CDS: coding sequence; EMSA: electrophoretic mobility shift assay; BR: brassinolide; CIPK: CBL-interacting protein kinase.
