# Supplementary material for: OsCAMTA3 Negatively Regulates Disease Resistance to Magnaporthe oryzae by Associating with OsCAMTAPL in Rice
Source: Int J Mol Sci. 2024 May 6;25(9):5049. doi: 10.3390/ijms25095049 (PMC11084498; doi:10.3390/ijms25095049)
Supplement: Supplementary file 1 [file ijms-25-05049-s001.zip › Supplementary Figures.pdf]

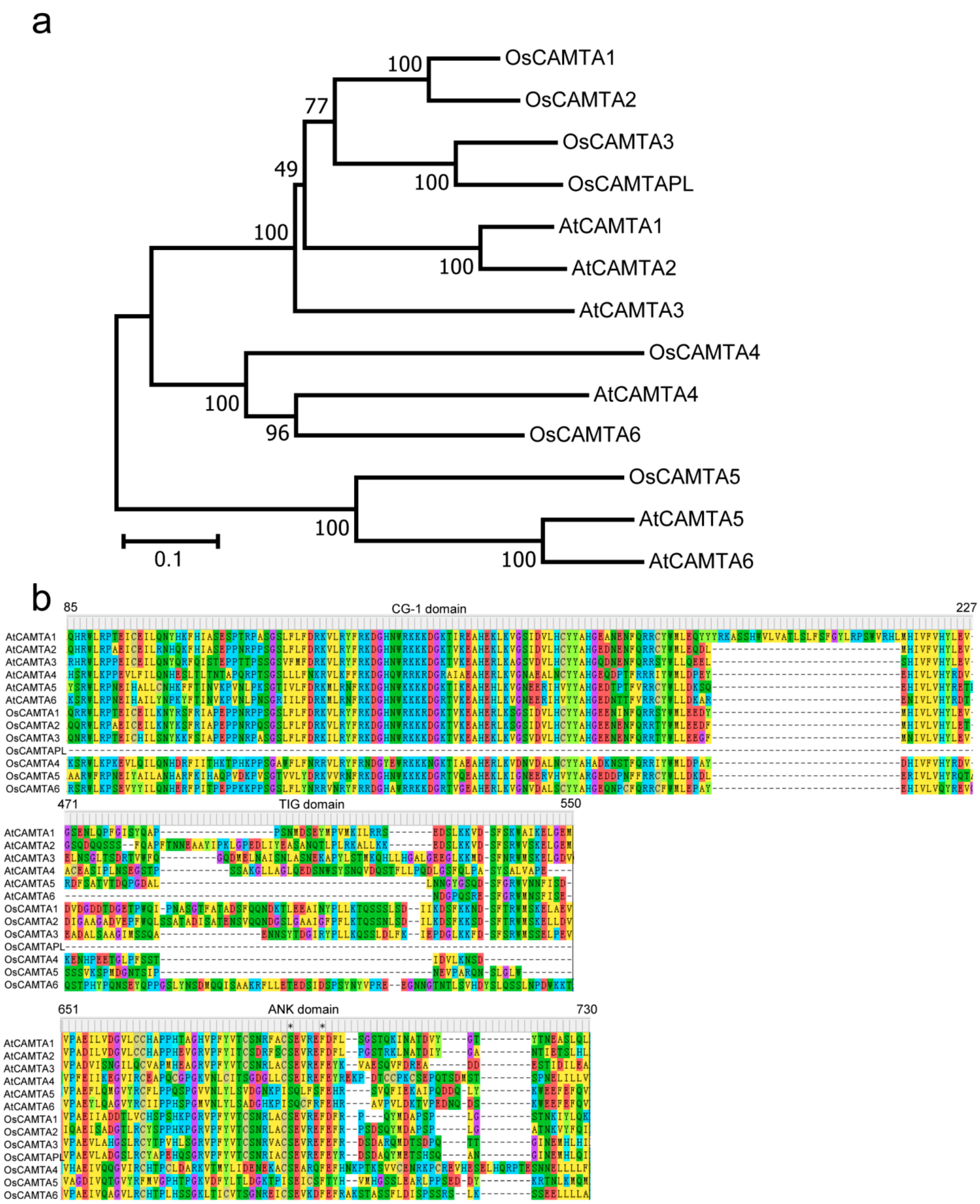

Figure S1. Evolutionary relationships of CAMTA between rice and Arabidopsis.(**a**) The evolutionary history was inferred using the neighbor-joining method. Proteins with a sum of branch length = 3.55009640 are shown. The percentage of replicate trees in which the associated taxa clustered together in the bootstrap test (500 replicates) is shown next to the branches. The analysis involved 13 amino acid sequences, and all positions containing gaps and missing data were eliminated. There were a total of 419 positions in the final dataset. Evolutionary analyses were conducted in MEGA7.0; (b) Alignment of the CG-1 domain and TIG-ANK domain of CAMTA homologs. The sequences were aligned using MEGA7.0.

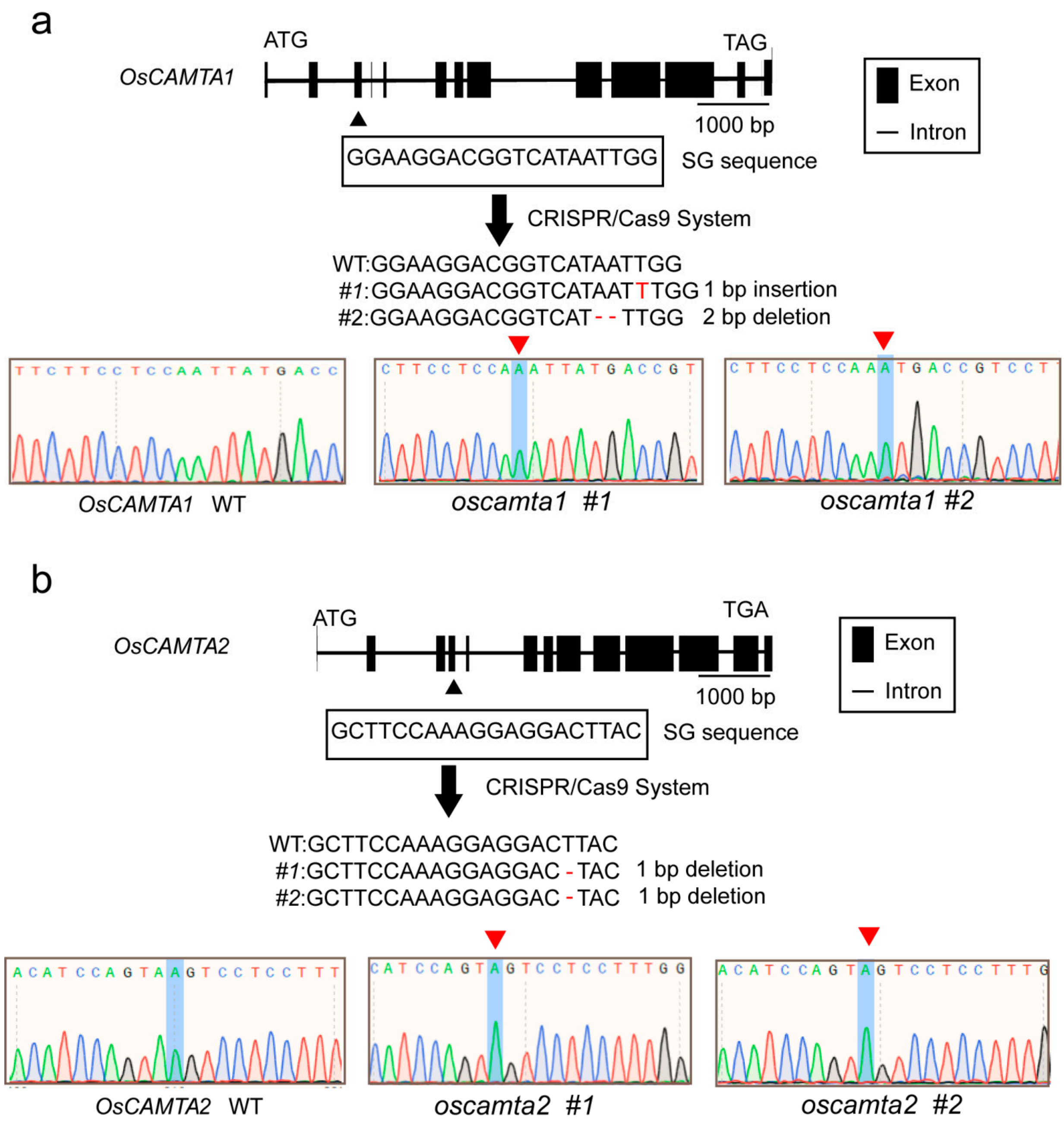

Figure S2. Schematic description of the CRISPR constructs for *OsCAMTA1/2*.Schematic description of CRISPR constructs and Sanger sequencing chromatograph of the genomic antisense sequences of *OsCAMTA1* (a) and *OsCAMTA2* (b). Black boxes indicate exons and lines indicate introns. The location of target sequences and mutations detected in genomic DNA sequences are shown. Two independent *oscamta1/2* double knockout mutants *oscamta1/2-1* and *oscamta1/2-2* contained a T insertion and a 2-bp TT deletion, respectively, in the *OsCAMTA1* coding region; and a T deletion in the *OsCAMTA2* coding region. The red triangles indicate the edited sites.

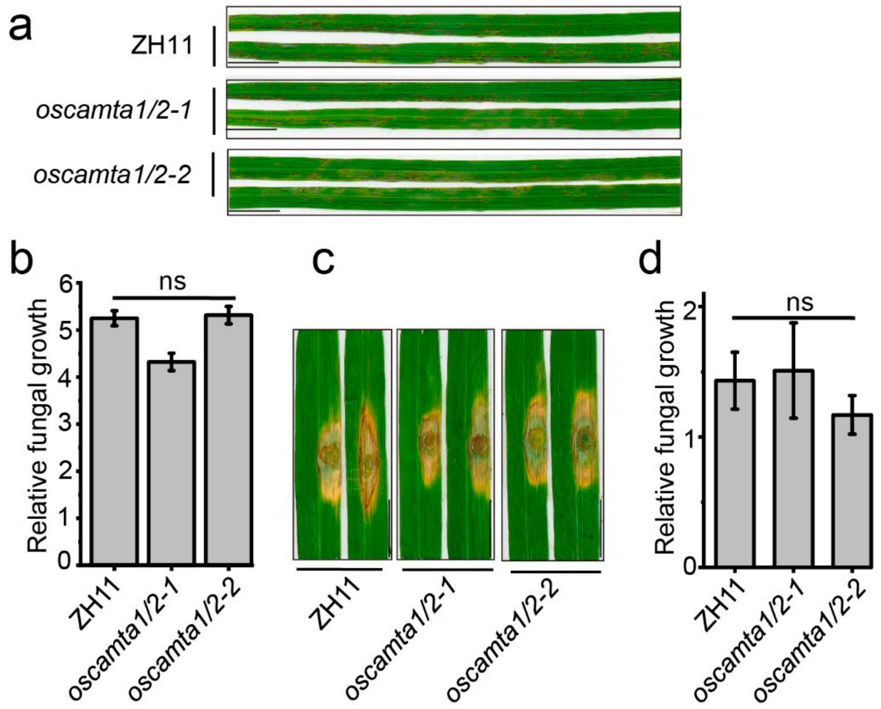

Figure S3. *oscamta1/2* exhibited no significant differences in disease resistance to *M. oryzae*. (a) 15-day-old ZH11 and *oscamta1/2* seedlings inoculated with *M. oryzae* by spraying. Images were taken at 5 dpi. Bar = 1 cm; (b) Fungal biomass of spraying-inoculated leaves was measured to quantify relative fungal growth in ZH11 and *oscamta1/2*. Data are presented as the means ± SEs (n = 3). ns means no significant difference. ($P < 0.05$; one-way ANOVA); (c) 25-day-old ZH11 and *oscamta1/2* seedlings inoculated with *M. oryzae* by punch inoculation. Images were taken at 7 dpi. Bar = 1 cm; (d) Fungal biomass of punch-inoculated leaves was measured to quantify relative fungal growth in ZH11 and *oscamta1/2*. Data are presented as the means ± SEs (n = 3). ns means no significant difference. ($P < 0.05$; one-way ANOVA).

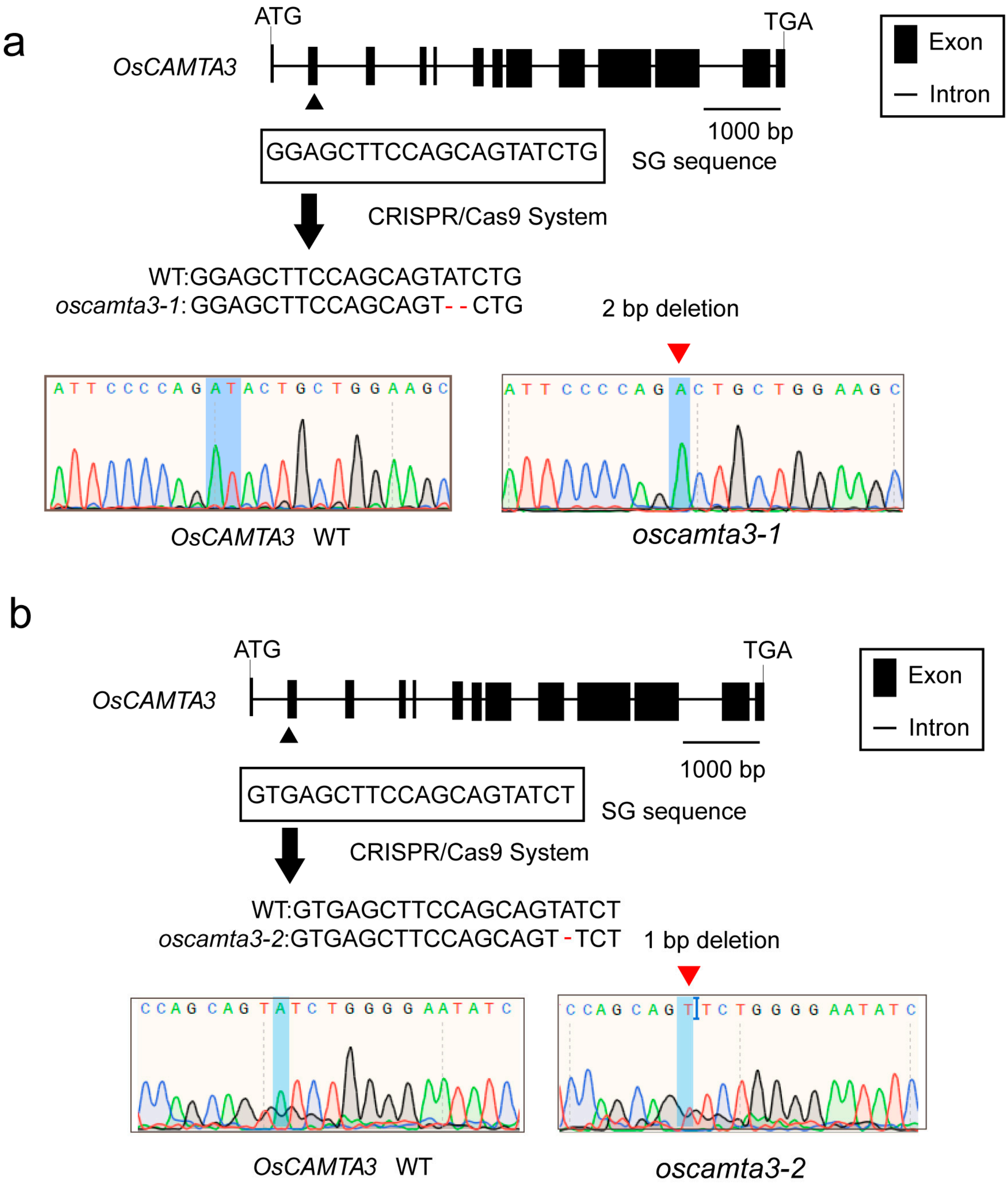

Figure S4. Schematic description of the CRISPR constructs for *OsCAMTA3*.Schematic description of CRISPR constructs and Sanger sequencing chromatograph of the genomic sequences of *OsCAMTA3*. Black boxes indicate exons and lines indicate introns. The location of target sequences and mutations detected in genomic DNA sequences are shown. Two independent *oscamta3* knockout mutants *oscamta3-1* and *oscamta3-2* contained a 2-bp AT deletion and an A deletion, respectively, in the *OsCAMTA3* coding region. The red triangles indicate the edited sites.

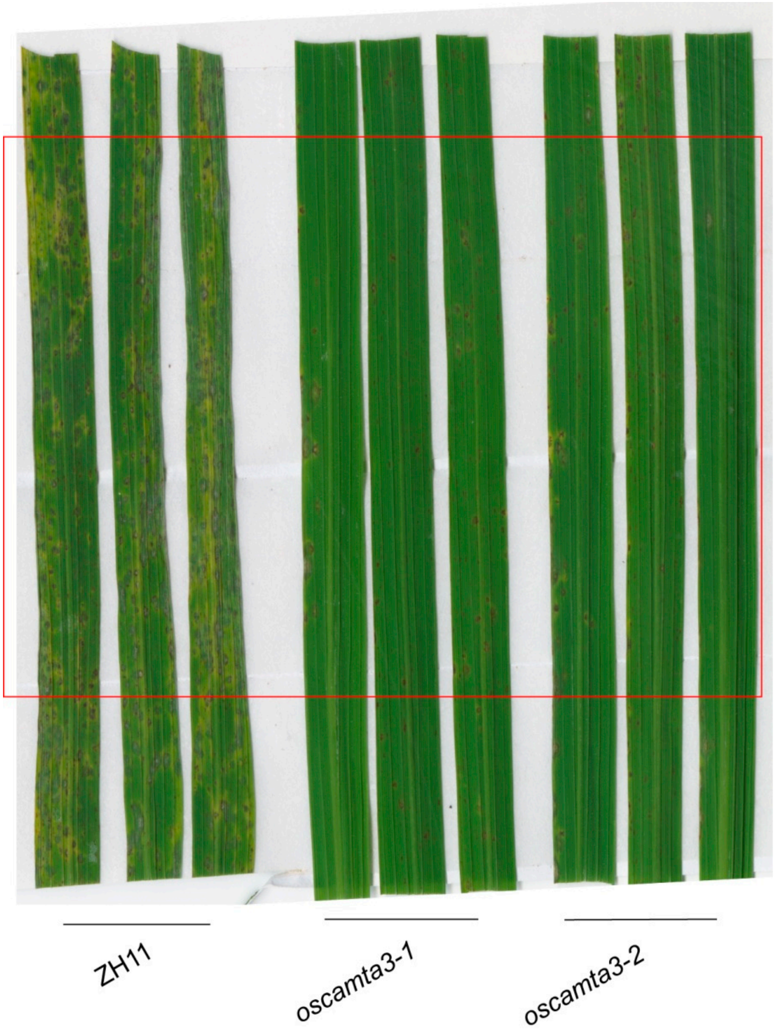

Figure S5. The uncropped rice leaves in Figure 1a. Fifteen-day-old ZH11 and *oscamta3* seedlings inoculated with *M. oryzae* by spraying. Images were taken at 5 dpi. This image is related to Figure 1a.

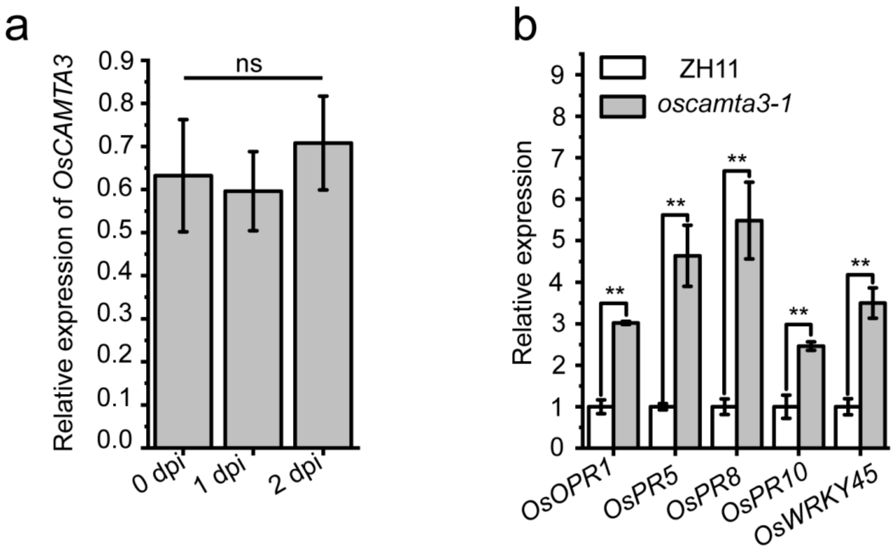

Figure S6. Relative expression of *OsCAMTA3* and *PR* genes. (a) The transcript accumulation of *OsCAMTA3* was examined by quantitative qRT–PCR. Data are presented as means ± SEs (n = 3). ns means no significant difference. (P<0.05; one-way ANOVA); (b) Relative expression of PR genes in ZH11 and *oscamta3-1*. Data are presented as means ± SEs (n = 3). (**; P < 0.01; Student's t-test).

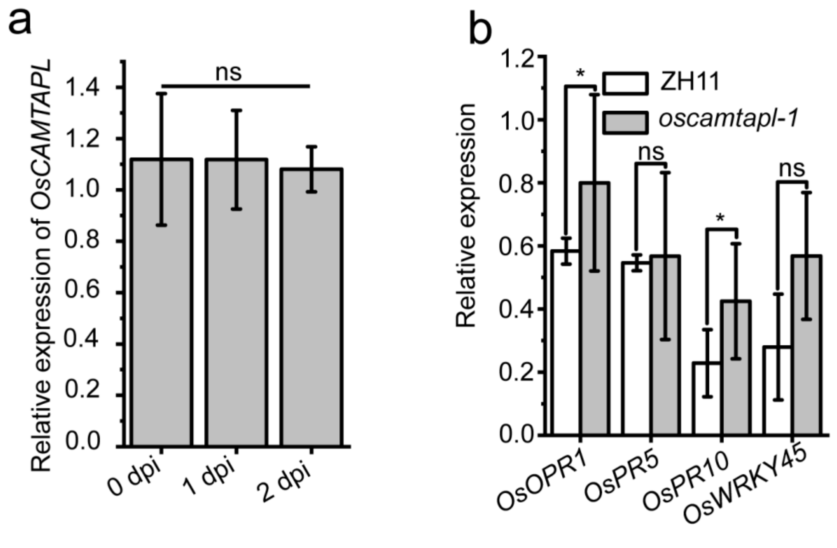

Figure S7. Relative expression of *OsCAMTAPL* and *PR* genes. (a) The transcript accumulation of *OsCAMTAPL* was examined by quantitative qRT–PCR. Data are presented as means ± SEs (n = 3). ns means no significant difference. ($P < 0.05$; one-way ANOVA); (b) Relative expression of *PR* genes in ZH11 and *oscamtapl-1*. Data are presented as means ± SEs (n = 3). ns means no significant difference. (*; $P < 0.05$; Student's *t*-test).

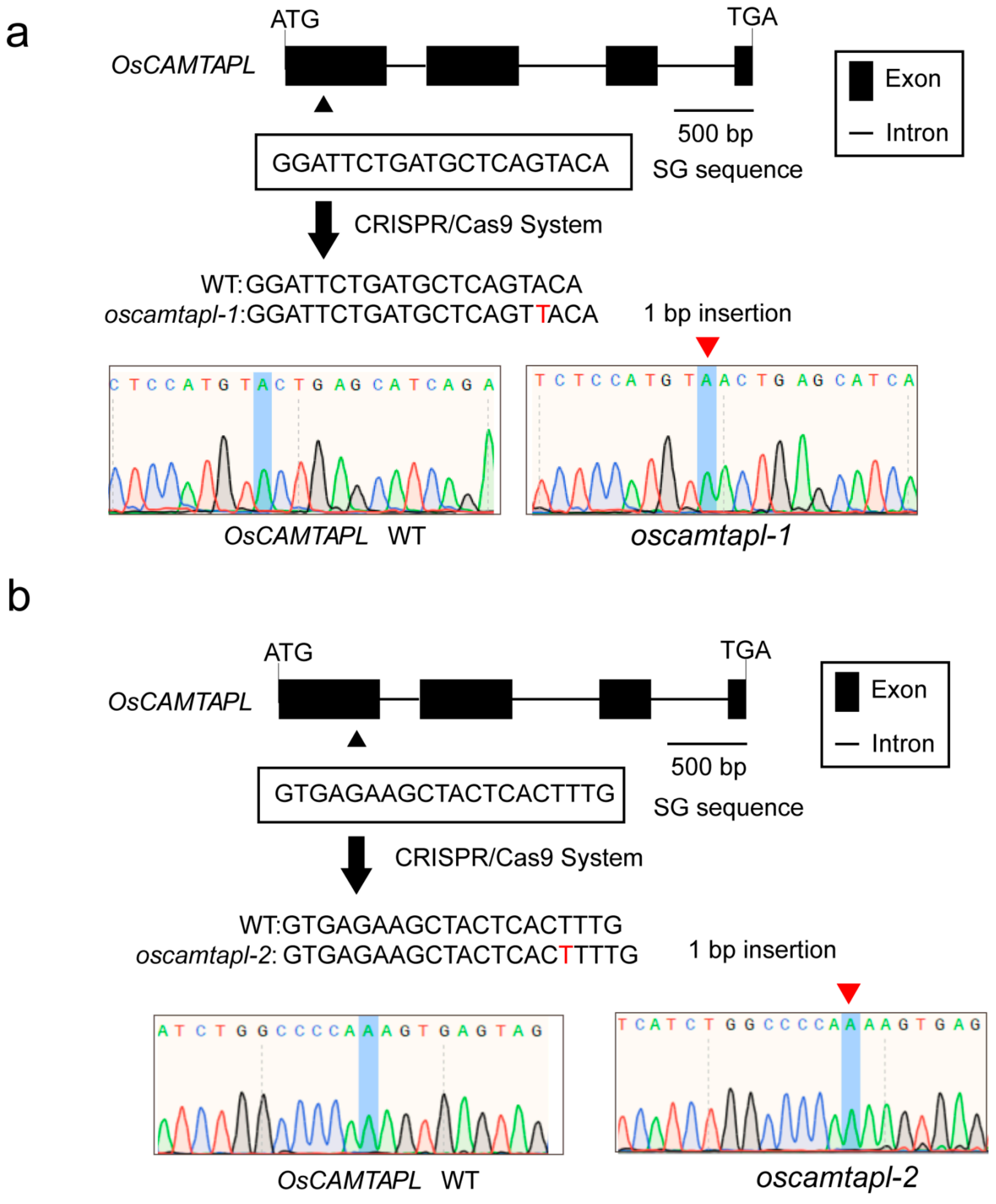

Figure S8. Schematic description of the CRISPR constructs for *OsCAMTAPL*.Schematic description of CRISPR constructs and Sanger sequencing chromatograph of the genomic sequences of *OsCAMTAPL*. Black boxes indicate exons and lines indicate introns. The location of target sequences and mutations detected in genomic DNA sequences are shown. Two independent *oscamtapl* knockout mutants *oscamtapl-1* and *oscamtapl-2* contained a T insertion in two different SG sequences, respectively, in the *OsCAMTAPL* coding region. The red triangles indicate the edited sites.

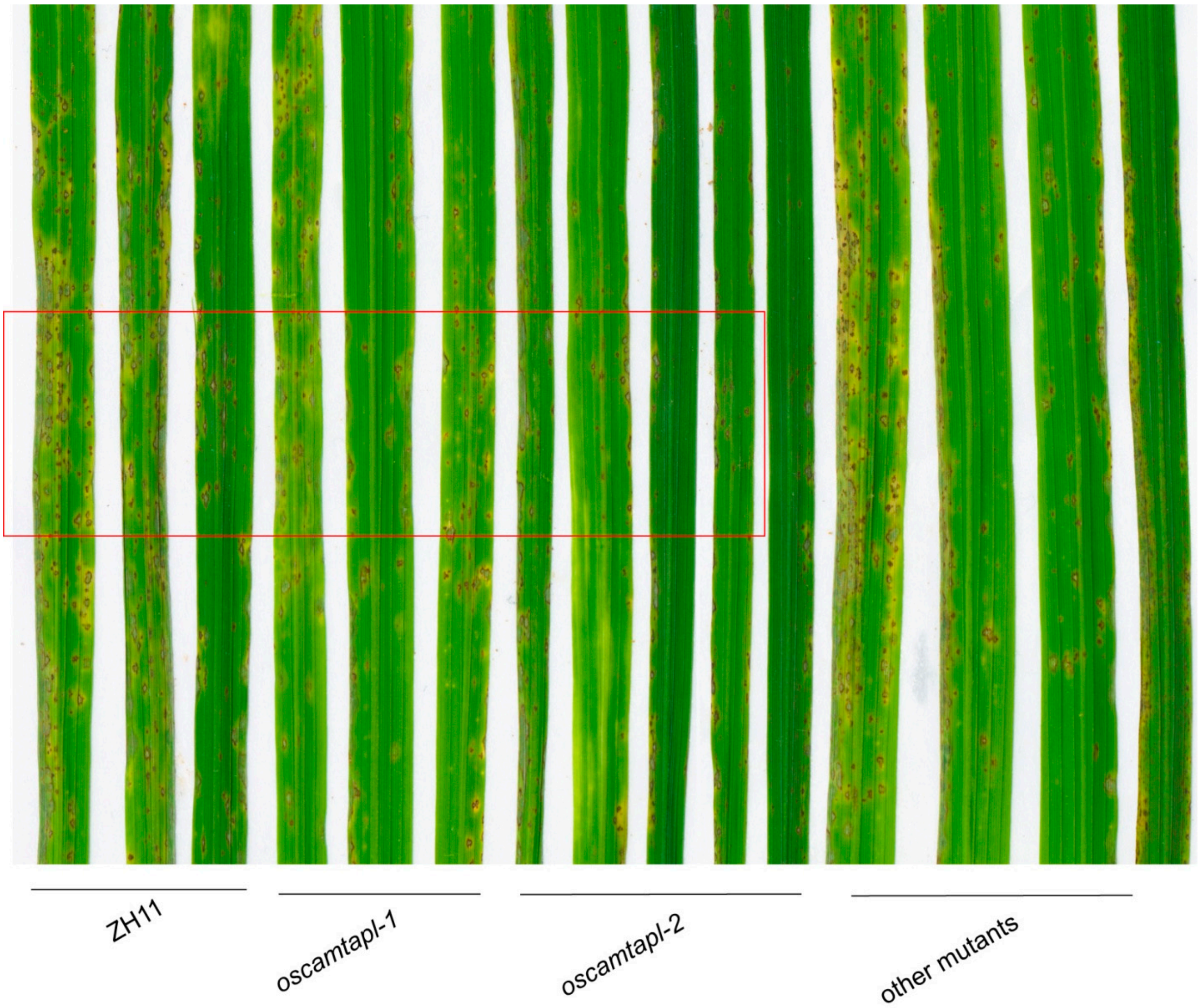

Figure S9. The uncropped rice leaves in Figure 2a. Fifteen-day-old ZH11 and *oscamtapl* seedlings inoculated with *M. oryzae* by spraying. Images were taken at 5 dpi. This image is related to Figure 2a.

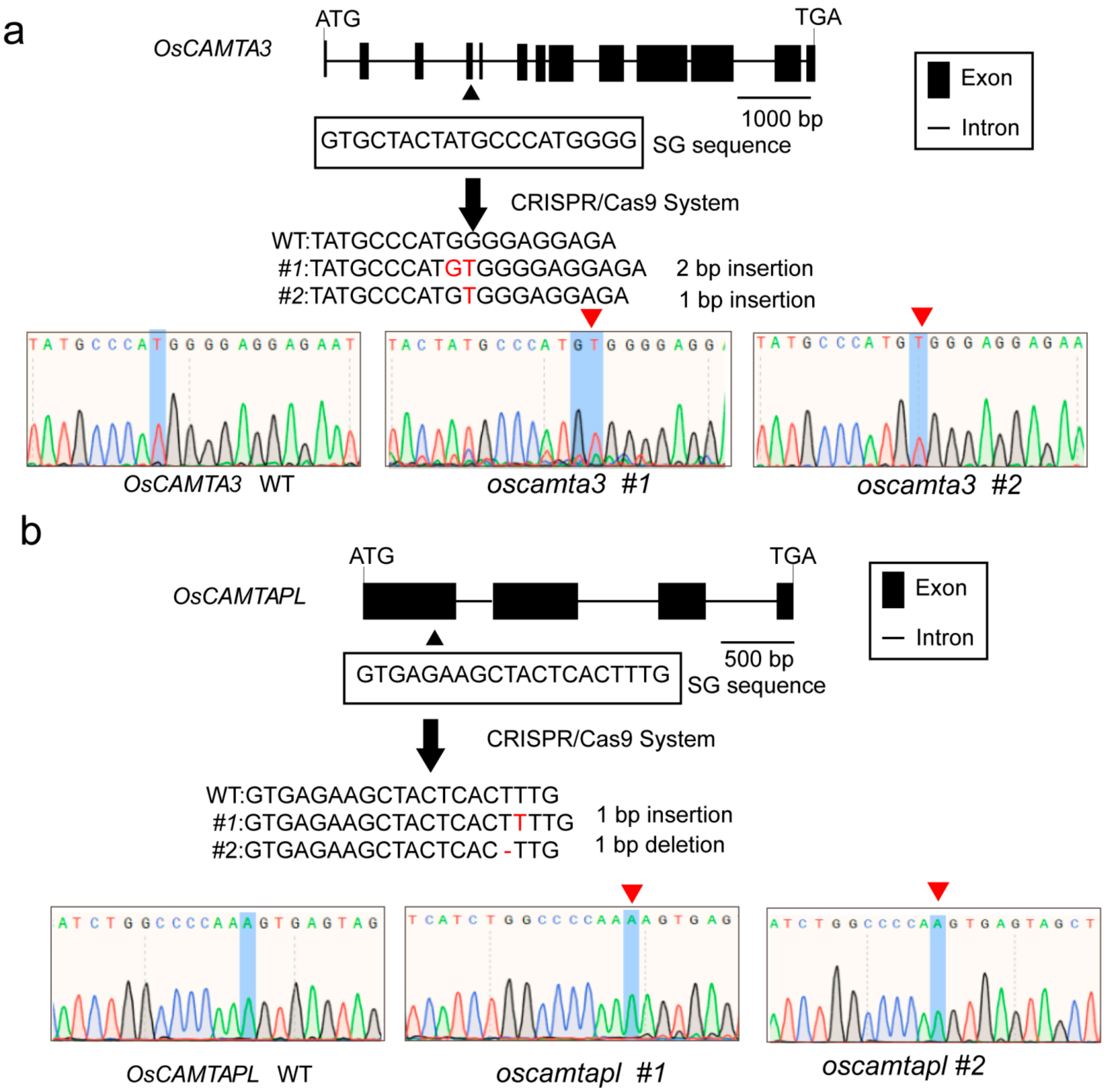

Figure S10. Schematic description of the CRISPR constructs for *OsCAMTA3* and *OsCAMTAPL* in *oscamta3/pl* double mutants.Schematic description of CRISPR constructs and Sanger sequencing chromatograph of the genomic anti-sense sequences of *OsCAMTA3* (a) and *OsCAMTAPL* (b) Black boxes indicate exons and lines indicate introns. The location of target sequences and mutations detected in genomic DNA sequences are shown. Two independent *oscamta3/pl* double knockout mutants *oscamta3/pl-1* and *oscamta3/pl-2* contained a 2-bp GT insertion and a T insertion, respectively, in the *OsCAMTA3* coding region; and a T insertion and a T deletion, respectively, in the *OsCAMTAPL* coding region. The red triangles indicate the edited sites.

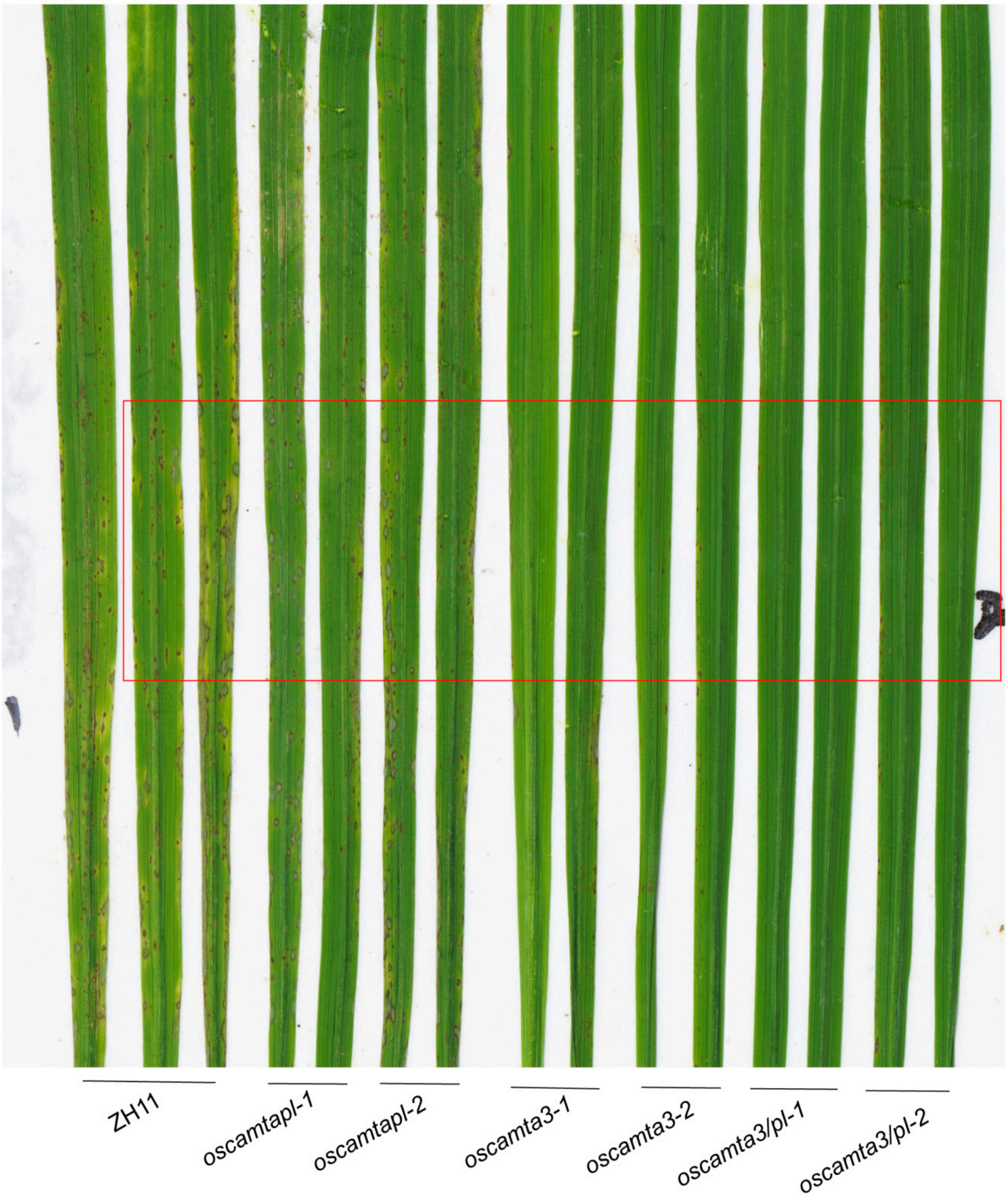

Figure S11. The uncropped rice leaves in Figure 3a. Fifteen-day-old ZH11, *oscamta3*, *oscamtapl*, and *oscamta3/pl* seedlings inoculated with *M. oryzae* by spraying. Images were taken at 5 dpi. This image is related to Figure 3a.

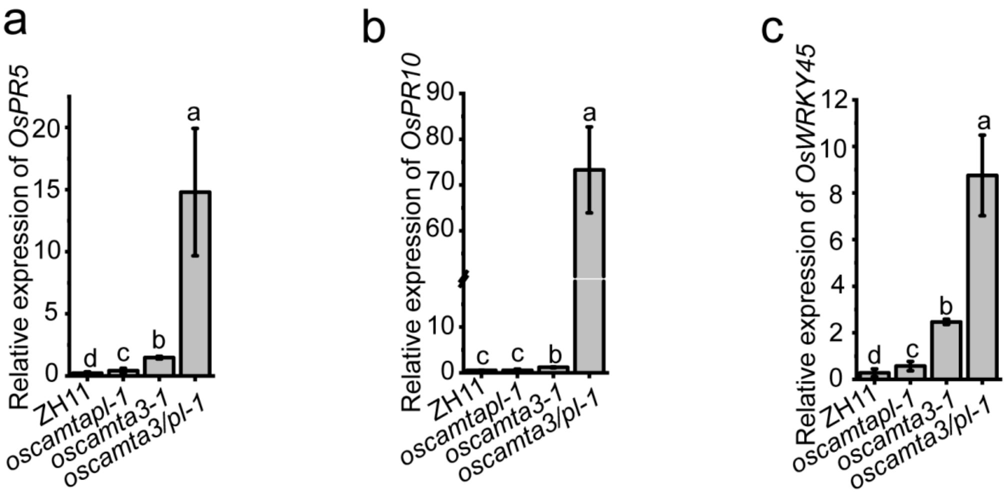

Figure S12. Detection of *PR* genes between *oscamtas* mutants. (a-c) Relative transcript levels of *PR* genes in ZH11 and high-order *OsCAMTAs* mutants. After the plants were sprayed with Guy11 conidia, the results were analyzed via qRT–PCR. Data are presented as means ± SEs (n = 3). Lowercase letters indicate statistically significant differences ($P < 0.05$; one-way ANOVA).

Co-ip uncropped full-length blots

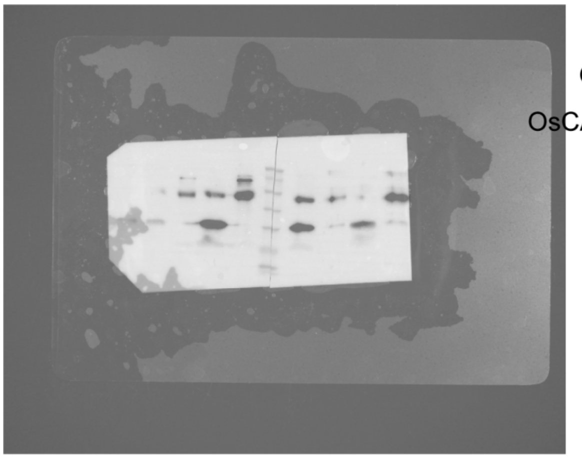

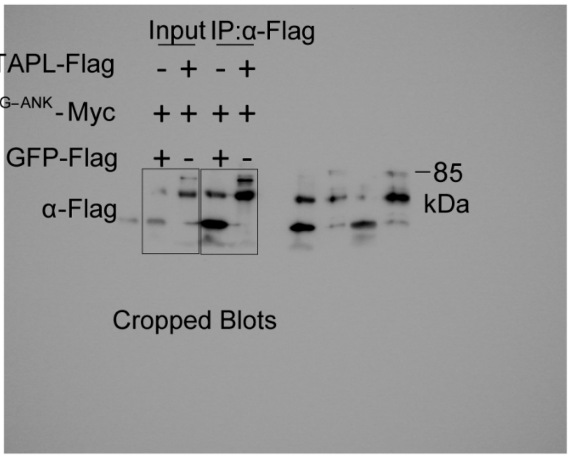

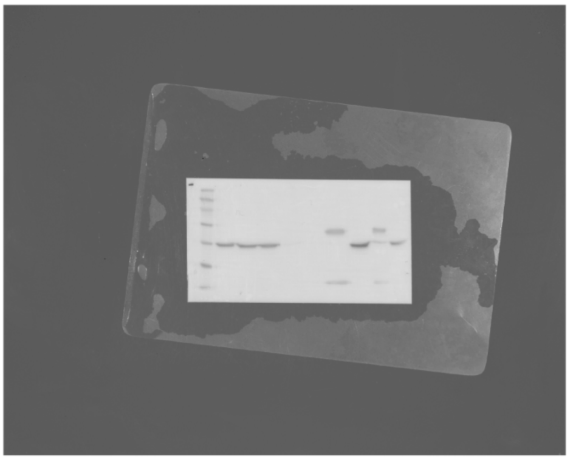

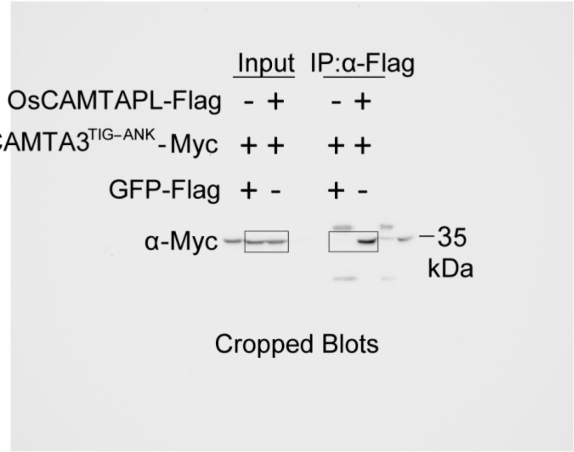

Figure S13. The uncropped full-length blots for co-IP assays in Figure 4d. co-IP assay was performed by transiently coexpressing OsCAMTA3$^{TIG-ANK}$-Myc and OsCAMTAPL-Flag in *N. benthamiana* leaves. Total protein was extracted and subjected to immunoprecipitation by anti-Flag beads. Immunoblotting analysis was performed with anti-Myc and anti-Flag antibodies. This image is related to Figure 4d.

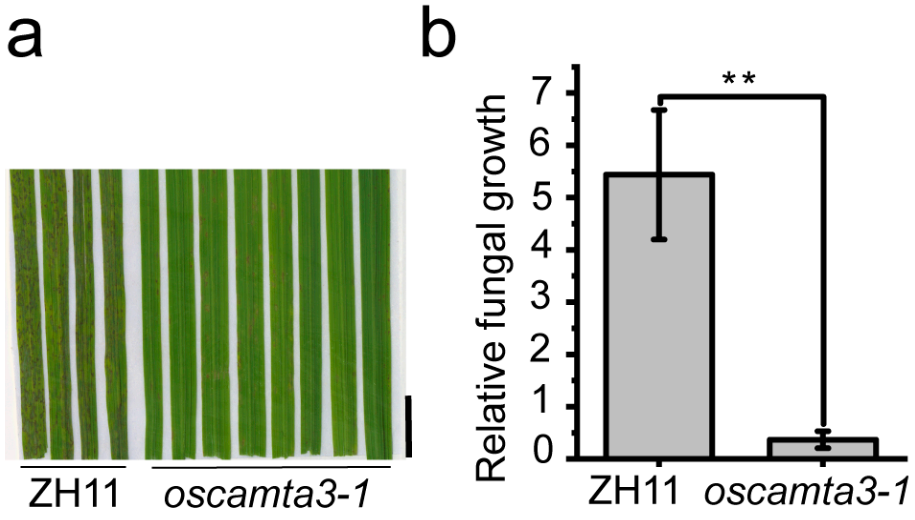

Figure S14. Material preparation for RNA-seq.(a) The *oscamta3-1* showed increased disease resistance compared with ZH11. Images were taken at 3 dpi; (b) Fungal biomass of spraying-inoculated leaves was measured to quantify relative fungal growth in ZH11 and *oscamta3-1*. Data are presented as the means ± SEs (n = 3). (**; $P < 0.01$; Student's t-test).

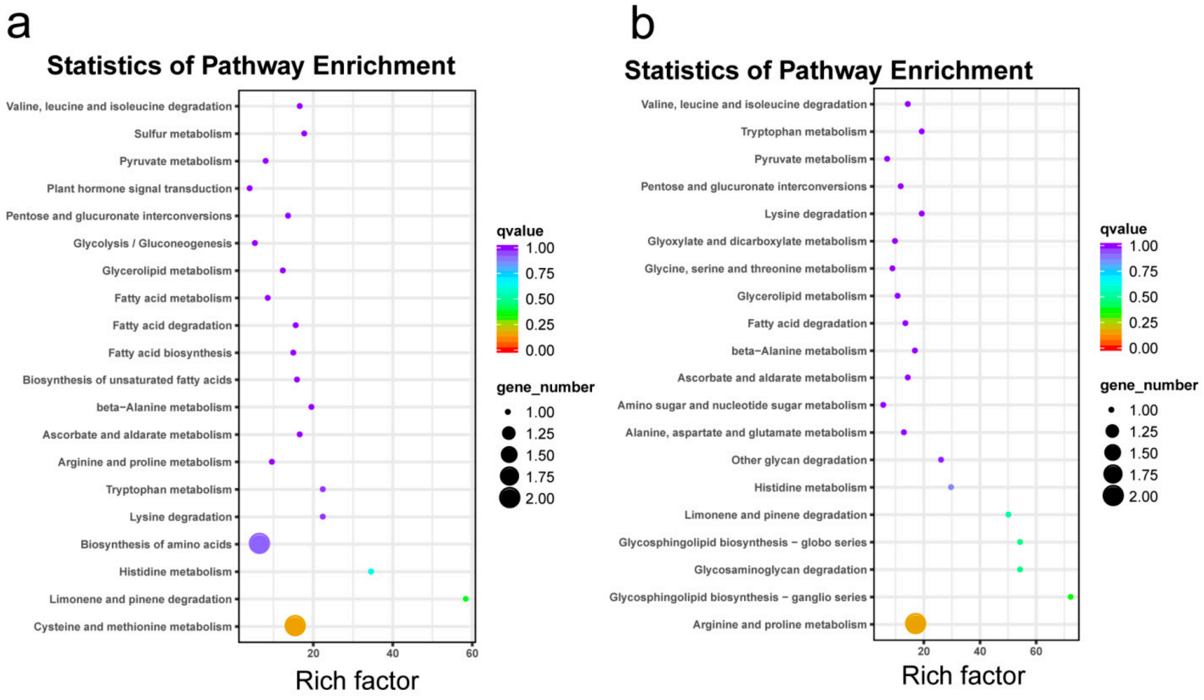

Figure S15. KEGG enrichment analyses of DEGs in ZH11 and *oscamta3-1* by RNA-seq. (a) KEGG enrichment analyses using the DEGs identified in ZH11 and *oscamta3-1* before inoculation with *M. oryzae*; (b) KEGG enrichment analyses using the DEGs identified in ZH11 and *oscamta3*-1 after inoculation with *M. oryzae.*

EMSA

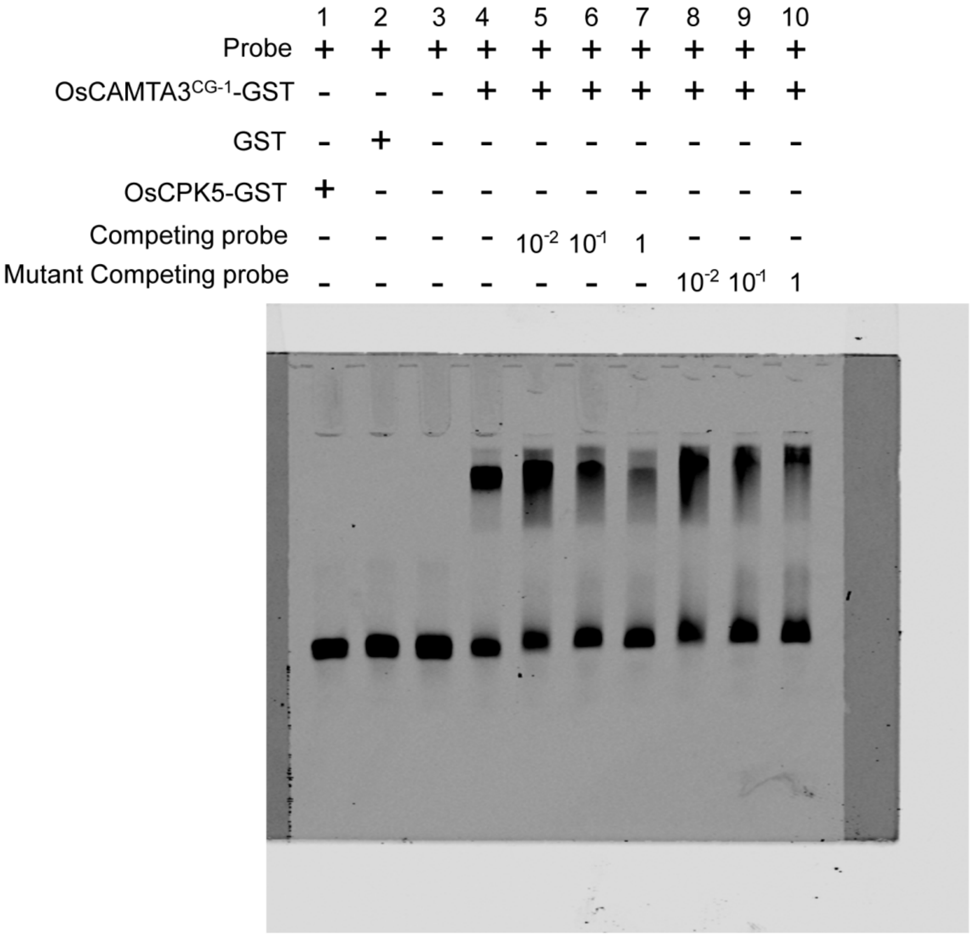

Figure S16. The uncropped full-length gel for the EMSA in Figure 8a.OsCAMTA3[CG-1] bound to the DNA fragment of the *OsALDH2B1* promoter containing the CGCG motif in an EMSA. This image is related to Figure 8a.

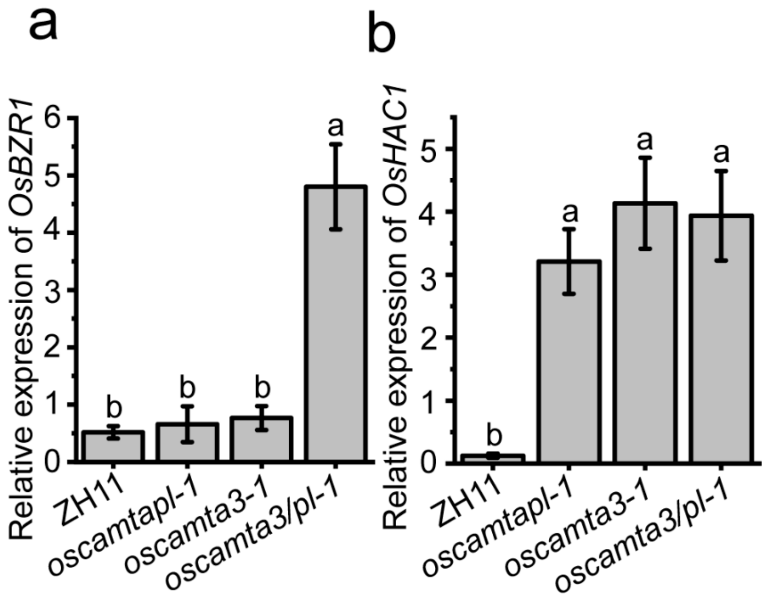

Figure S17. Detection of the transcription of *OsBZR1* and *OsHAC1* between ZH11 and *oscamtas* mutants.Data are presented as the means ± SEs (n = 3). Lowercase letters represent statistically significant differences ($P < 0.05$; one-way ANOVA).
